# A quadratic high-gain DC–DC converter integrating a two-winding coupled inductor with low voltage stress on both switches for renewable energy applications

**DOI:** 10.1038/s41598-026-49207-6

**Published:** 2026-04-28

**Authors:** Ali Nadermohammadi, Seyed Hossein Hosseini, Mehran Sabahi

**Affiliations:** 1 Faculty of Electrical and Computer Engineering, University of Tabriz, Tabriz, 51666-16471 Iran; 2 Engineering Faculty, Near East University, Nicosia, 99138 Turkey

**Keywords:** Energy science and technology, Engineering

## Abstract

This paper introduces a quadratic, high-gain DC–DC converter tailored for renewable-energy interfaces. The architecture marries a quadratic step-up stage with a two-winding coupled inductor (CI) to deliver a large voltage boost. Voltage gain is tunable via two independent knobs the switches’ duty cycle and the CI turns ratio giving the designer extra freedom. Hallmark features include very high gain, low voltage stress on the switches, continuous input current, a shared ground at input and output, high efficiency, and synchronized gate drive. We detail the operating modes, steady-state characteristics, design rules, and efficiency metrics, and we provide dynamic modeling with control analysis. To showcase its benefits, the proposed converter is benchmarked against related topologies. Its practicality is validated on a 400-W prototype operating at 50 kHz, stepping 25 V up to 400 V.

## Introduction

**Fig. 1:**
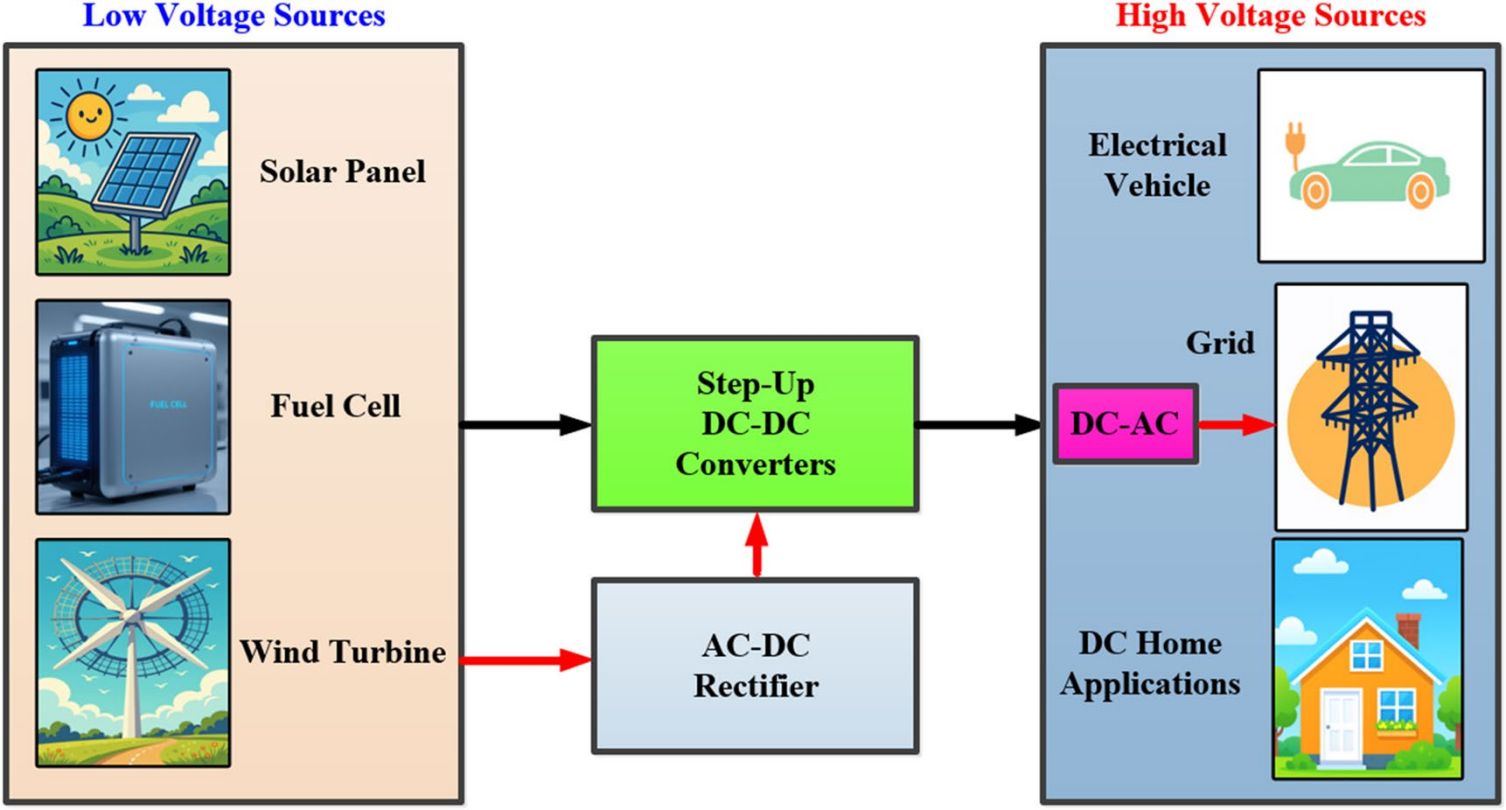
Energy flow from low-voltage sources to high-voltage applications.

Over the last ten years, global efforts have increasingly centered on cutting carbon emissions and expanding the role of renewable energy in the power sector. As illustrated in Fig. 1, renewable resources are now viewed as the most viable substitutes for conventional fossil fuels. Among these, photovoltaic (PV) technology has emerged as a front-runner because it is abundant, sustainable, and imposes relatively little harm on the environment. A major technical challenge, however, is that PV panels and fuel cells generate direct current (DC) at relatively low voltage levels, which cannot be connected directly to the power grid. To overcome this limitation, high-gain DC–DC boost converters are employed. These devices elevate the modest voltage produced by renewable sources to levels appropriate for subsequent processing and integration^1,2^. In this way, they serve as critical components in renewable energy systems, enabling cleaner electricity generation and supporting global efforts to curb air pollution, mitigate ecological damage, and slow the progression of climate change driven by fossil fuel consumption.

To achieve high voltage gain and increase the low-level voltage of renewable energy sources such as PV systems, a wide range of specialized boosting techniques have been employed in step-up DC–DC converters^3^. These include switched-capacitor (SC) and switched-inductor (SL) networks, active switched-inductor (A-SL) configurations, coupled-inductor structures, voltage-lift and voltage-multiplier (VM) strategies, as well as interleaved and cascaded topologies^4–7^. In many designs, these methods are also combined to further enhance performance.

The switched-capacitor approach is widely preferred in step-up converter design because it can significantly enhance voltage gain while simultaneously lowering voltage stress^8^. Despite these benefits, achieving higher gain requires adding more SC cells, which increases conduction losses and reduces overall efficiency^8^. In addition, these converters often suffer from pronounced inrush currents during switching events, which place extra current stress on the power switches and degrade efficiency. Alternative methods include the SL and active A-SL techniques. Studies in^9,10^ have explored hybrid designs that combine SC and SL networks to achieve large voltage gains; however, these topologies demand a high number of components and typically lack a common ground connection. Another strategy for enhancing voltage gain in DC–DC converter designs is the use of multi-stage or multi-level topologies. These structures are generally categorized into three main types: cascaded, interleaved, and multilevel configurations^11^. Despite their effectiveness in achieving high gain, such converters often encounter drawbacks, including complex control requirements, a large component count, and increased implementation costs. Studies in^12,13^ propose interleaved converter structures as effective options for PV applications, mainly due to their capability to suppress input current ripples, which enhances suitability for such systems. Nevertheless, these converters are limited by the complexity of their control strategies and the relatively high implementation cost. Integrating boost techniques with one another, or with a CI stage, maximizes voltage gain while allowing operation at a low duty cycle, which cuts conduction loss^14–29^. References ^30–32^ introduce two high step-up Quasi-Z-Source (QZS) converters that employ a CI and operate with a two switch. These designs are capable of delivering considerable voltage gain, they suffer from a key limitation: the duty cycle cannot exceed 50%, which restricts their operation at higher duty ratios. In^33,34^, a high–conversion-ratio design is realized by combining coupled inductors with switched-capacitor connections on the secondary side. The principal limitation is pronounced ripple at the input current, making an input filter necessary. In ^35^, high-gain Dual-Duty-Triple-Mode (DDTM) converters arise from coupling a three-switch active switched-inductor stage with switched capacitors, allowing two independent duty commands. Drawbacks include floating input/output grounds and substantial input-current ripple. The solution in^36^ restores a common ground and suppresses ripple, though it requires an exceptionally high duty ratio on the second switch to reach the desired gain. In ^37,38^, three-port topologies using coupled inductors are proposed to deliver multiple required output voltages. While very high voltage gain is obtained via high CI turns ratios, the resulting efficiency is poor. Moreover, both output ports share the same control path, so neither is independently driven. Reference^39^ presents a three-port converter that integrates a quadratic structure with a coupled inductor, achieving ultra-high gain with a modest turns ratio and small duty cycle thereby raising efficiency but it likewise does not provide independent switching control for the two outputs. Integrating a CI with quadratic-based and boost-cell techniques, as in^40–53^, yields substantial voltage gain; however, these converters suffer from low efficiency due to pronounced semiconductor losses. In^44^, a quadratic converter with low device stress is proposed; despite its large conversion ratio, the series connection between the CI primary and the input source produces pronounced input-current ripple, which burdens the source. Work^45^ reports high-gain converters with low input-current ripple by pairing a coupled inductor implemented in a dual-secondary arrangement with voltage-multiplier cells. The added degrees of freedom allow broad tuning of the conversion ratio, but the topology suffers from high voltage stress on the main switch and heavy current stress on the input diodes. Reference^46^ advances a quadratic DC–DC design that combines a CI with a SC network to realize high gain; however, peak capacitor currents are large, leading to elevated losses and increased EMI. In^47^, a quadratic impedance-source scheme integrated with a CI attains high step-up capability using few devices, yet imposes substantial voltage stress on the power semiconductors. The quadratic boost SEPIC architecture analyzed in^48^ achieves high gain with low input ripple beneficial for DC-microgrid use but its additional passive elements, especially the coupling capacitor, introduce resistive/reactive losses that depress efficiency. Finally^49^, presents a high step-up quadratic converter with continuous input current and reduced semiconductor stress; the trade-off is a large parts count particularly magnetic cores which lowers power density and raises cost.

Motivated by the strengths and limitations of existing high step-up converters, this paper proposes a quadratic, high-gain DC–DC architecture tailored for renewable-energy applications. The proffered configuration offers the following merits:

1. Voltage gain is tunable via two independent parameters the power-switch duty cycle and the CI turns ratio providing flexible gain control during design and optimization.
2. The maximum voltage stress is small (≈ 12.5% of V_out_ ​ on S_1_ and ≈ 25% of V_out_ on S_2_), enabling low voltage, low R_DS_ (_on_) devices that cut conduction loss and improve efficiency.

3) The converter attains large output voltage with modest duty cycles, further reducing conduction losses in the switches.
4) The input current is continuous with small ripple, easing source stress and input-filter requirements.
5) Only two active switches are utilized, driven in synchrony for simplified control.
6) A shared ground reference helps suppress common-mode emissions compared with non-common-ground designs, reducing EMI concerns.

## Proposed converter and operation modes

**Fig. 2:**
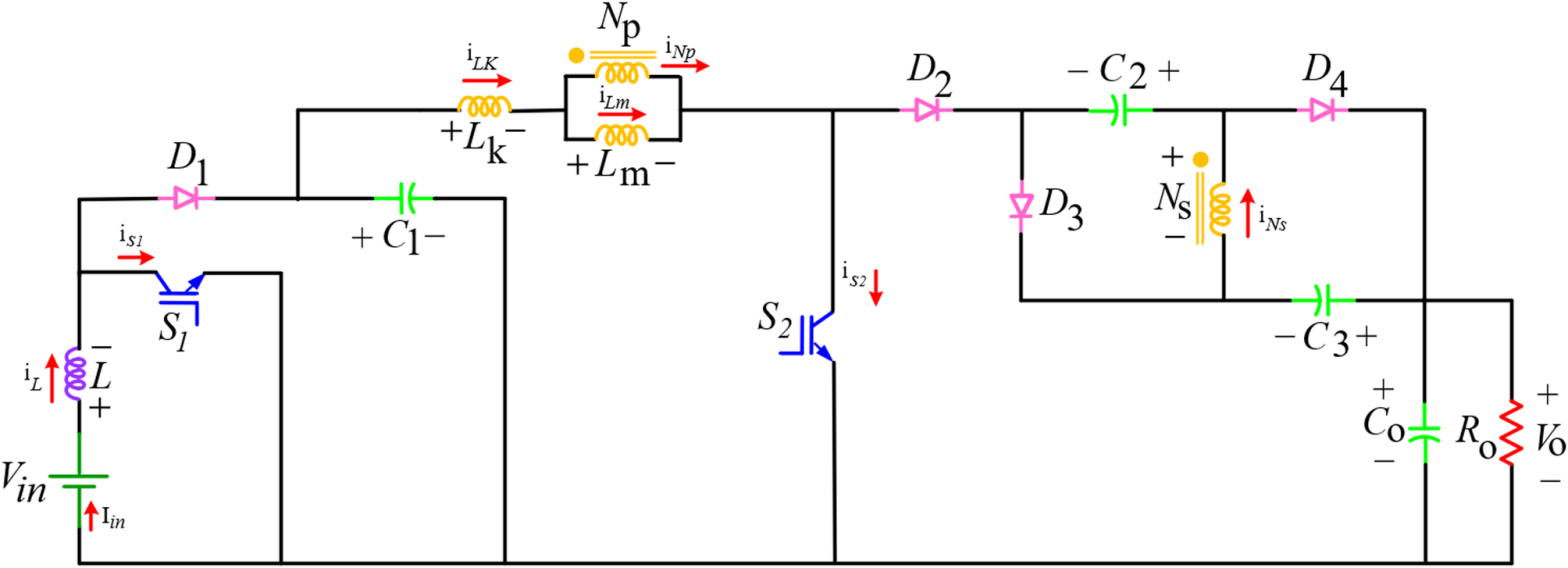
Power circuit of the propounded design.

**Fig. 3:**
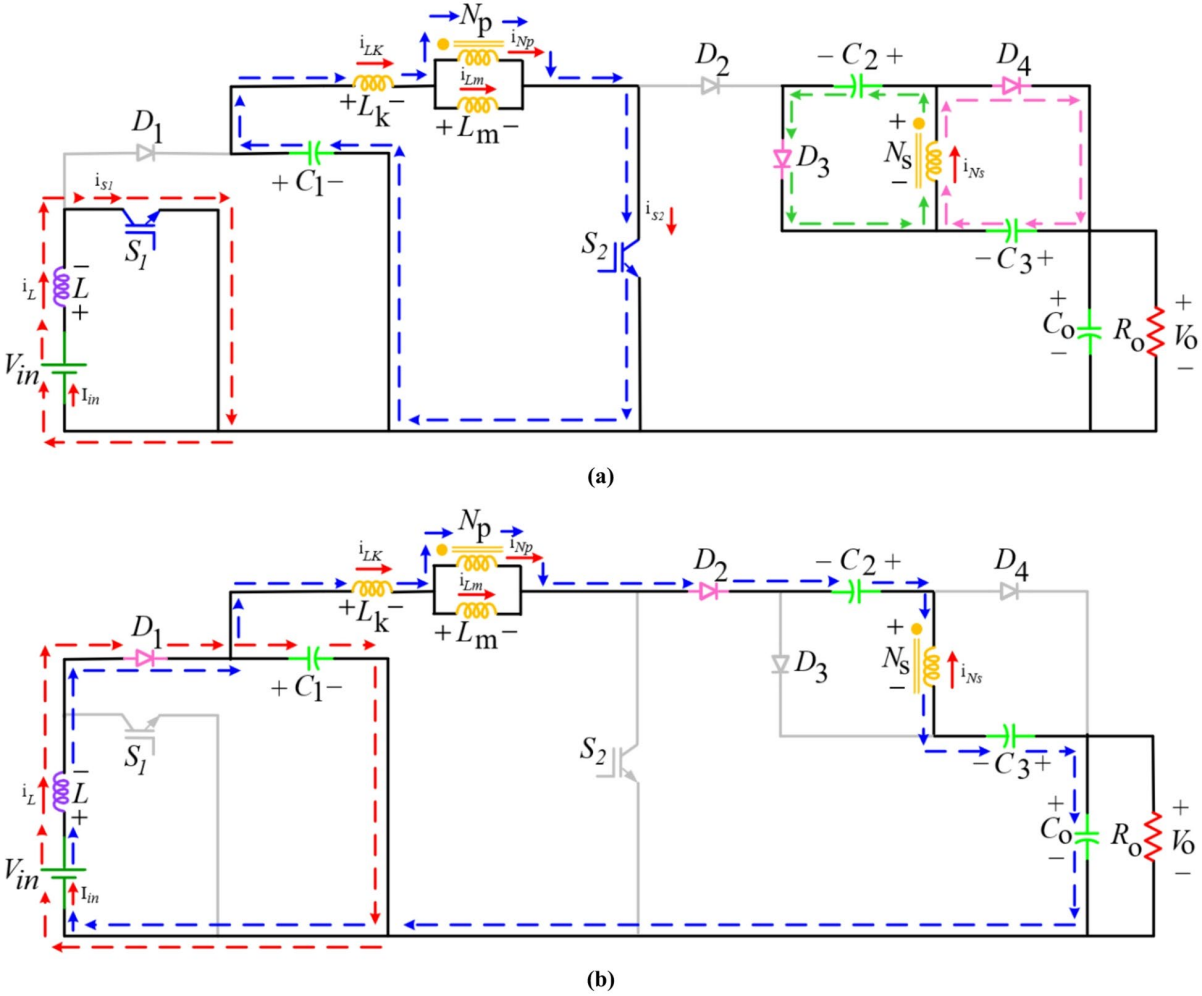
Equivalent circuits of the proffered design (**a**) First switching subinterval, (**b**) Second switching subinterval.

**Fig. 4:**
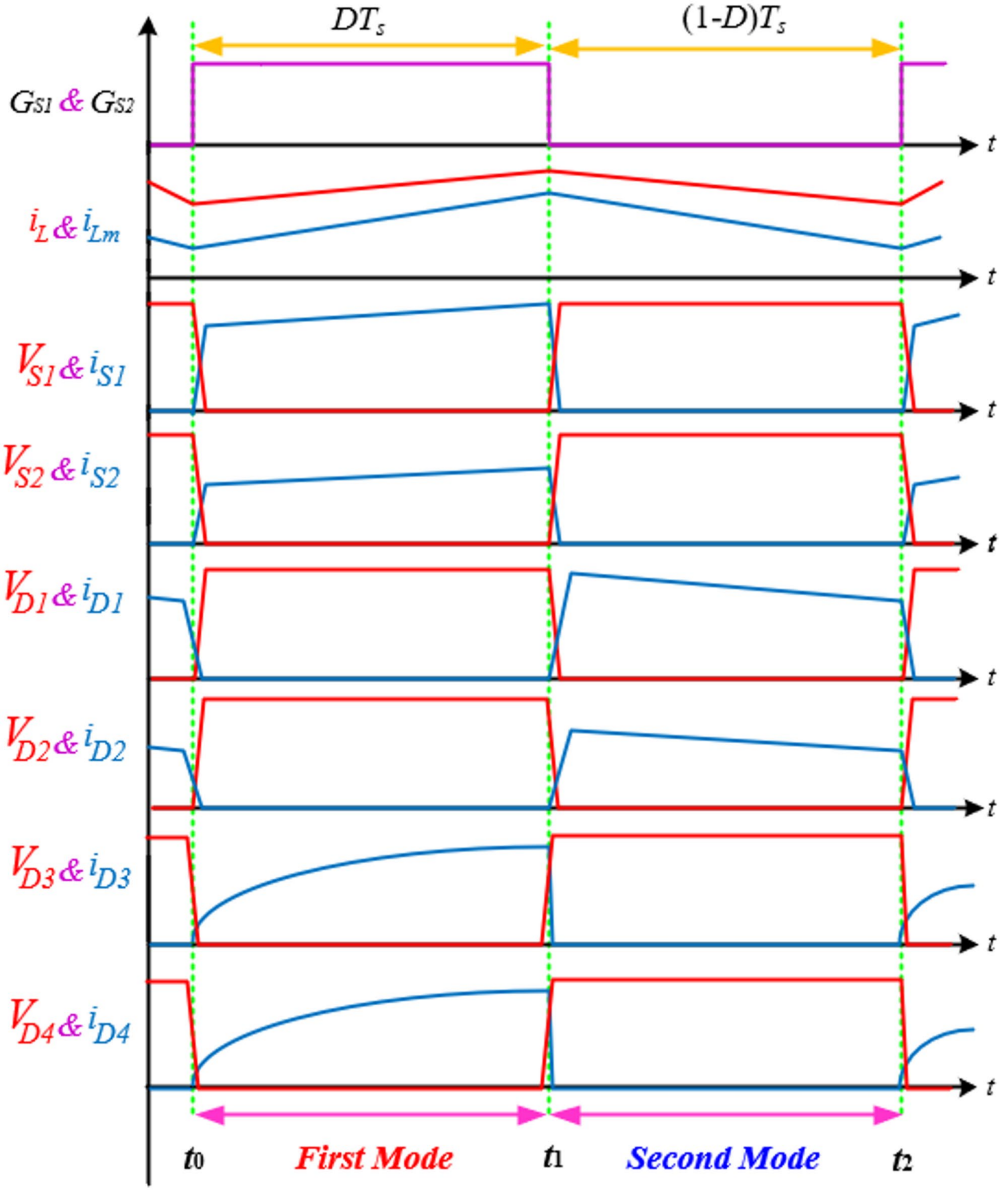
Main waveforms of the proffered circuit.

The proffered quadratic circuit shown in Fig. 2 outlines the structure of the suggested configuration. This circuit consists of an input inductor (L), two switches (S_1_ and S_2_), four capacitors (C_1_, C_2_, C_3_, and C_O_), four diodes (D_1_ to D_4_), and a coupled inductor arranged with two windings: a primary winding (N_P_) and a secondary winding (N_S_). Additionally, the turns ratio of the coupled inductor is defined as n = N_S_ / N_P_, while the coupling coefficient is given by k = L_m_ / (L_m_ + L_k_). The circuit operates in continuous conduction mode (CCM) and can be divided into two distinct subintervals, as shown in Fig. 3. The main voltage and current waveforms for these components are presented in Fig. 4.

### First switching subinterval

During this operating interval, both switches are turned on, causing diodes D_3_ and D_4_ to conduct forward, while the other diodes remain reverse-biased. The magnetizing inductance L_m_ is energized through the path formed by C_1_, L_m_, and switch S_2_, which leads to a linear rise in the current i_Lm_ and simultaneous discharge of capacitor C_1_. Meanwhile, the input inductor L is charged via the path consisting of V_in_, L, and switch S_1_, producing a linear increase in current i_L_. Concurrently, capacitor C_2_ is charged through the loop containing C_2_, diode D_3_, and the secondary winding N_S_, while capacitor C_3_ is charged through the path including C_3_, N_S_, and diode D_4_. Throughout this stage, the circuit behavior adheres to the constraints set by Kirchhoff’s Voltage Law (KVL) and Kirchhoff’s Current Law (KCL).

1 $${V_{Ns}}=n{V_{Lm}}$$

2 $${V_1}={V_{Lm}}+{V_{Lk}}=\frac{{{V_{Lm}}}}{k}$$

3 $${V_{in}}={V_L}$$

4 $${V_1}={V_{C1}}$$

5 $${V_{C2}}={V_{Ns}}$$

6 $${I_{Np}}=n{I_{Ns}}$$

7 $${I_L}={I_{in}}$$

8 $${I_L}={I_{S1}}$$

9 $${I_{C1}}= - {I_{Np}} - {I_{Lm}}$$

10 $${I_{S2}}={I_{Np}}+{I_{Lm}}$$

11 $${I_{C2}}={I_{D3}}$$

12 $${I_{D3}}={I_{Ns}} - {I_{C3}}$$

13 $${I_{Ns}}={I_{C2}}+{I_{D4}}$$

14 $${I_{Co}}={I_{D4}} - {I_O}+{I_{D3}} - {I_{Ns}}$$

15 $${I_{S1}}= - {I_{Co}} - {I_O} - {I_{S2}}+{I_{Np}}+{I_{Lm}}+{I_{in}}$$

16 $${I_{D4}}= - {I_{D3}}+{I_{Ns}} - {I_{S2}} - {I_{C1}} - {I_{S1}}+{I_{in}}$$

### Second switching subinterval

During this phase, both power switches are turned off, causing diodes D_1_ and D_2_ to conduct forward, while the other diodes remain inactive. The magnetizing inductance L_m_ discharges through the circuit path formed by V_in_, L, D_1,_ L_m_, D_2_, C_2_, N_S_, C_3_, and C_O_, leading to a gradual reduction in the current i_Lm_, while capacitors C_2_ and C_3_ discharge along this path. Simultaneously, the input inductor L discharges through the loop consisting of V_in_, L, D_1_, and C_1_, resulting in a linear decline in current i_L_ as capacitor C_1_ discharges along this path. The governing equations for this interval can be expressed as follows.

17 $${V_{in}}={V_L}+{V_{C1}}$$

18 $${V_{C1}}={V_1} - {V_{C2}}+{V_{Ns}} - {V_{C3}}+{V_O}$$

19 $${I_L}={I_{in}}$$

20 $${I_L}={I_{D1}}$$

21 $${I_{C1}}={I_{D1}} - {I_{Np}} - {I_{Lm}}$$

22 $${I_{D2}}={I_{Np}}+{I_{Lm}}$$

23 $${I_{C2}}= - {I_{D2}}$$

24 $${I_{C3}}={I_{Ns}}$$

25 $${I_{Ns}}={I_{C2}}$$

26 $${I_{Co}}= - {I_O} - {I_{Ns}}$$

27 $${I_{D1}}= - {I_{Co}} - {I_O}+{I_{Np}}+{I_{Lm}}+{I_{in}}$$

28 $${I_{in}}={I_{C1}} - {I_{Ns}}$$

### Voltage gain calculation

The volt-second balance principle for the inductor L and CI can be applied as follows:

29 $${\left\langle {{V_{Lm}}} \right\rangle _{{T_S}}}=0$$

30 $${\left\langle {{V_1}} \right\rangle _{{T_S}}}=0$$

31 $${\left\langle {{V_L}} \right\rangle _{{T_S}}}=0$$

The capacitor voltage expressions can be derived from the governing equations, as presented below:

32 $${V_{C1}}=\frac{{{V_{in}}}}{{1 - D}}$$

33 $${V_{C2}}={V_{C3}}=\frac{{{V_{in}}kn}}{{1 - D}}$$

The mathematical expression for the output voltage of the proposed circuit is given as:

34 $${V_O}=\frac{{{V_{in}}(2kn - Dkn+1)}}{{{{(1 - D)}^2}}}$$

By neglecting the effect of the coupling coefficient CI (particularly when k = 1), the voltage gain can be expressed as:

35 $$G=\frac{{{V_O}}}{{{V_{in}}}}=\frac{{2kn - Dkn+1}}{{{{(1 - D)}^2}}}$$

**Fig. 5:**
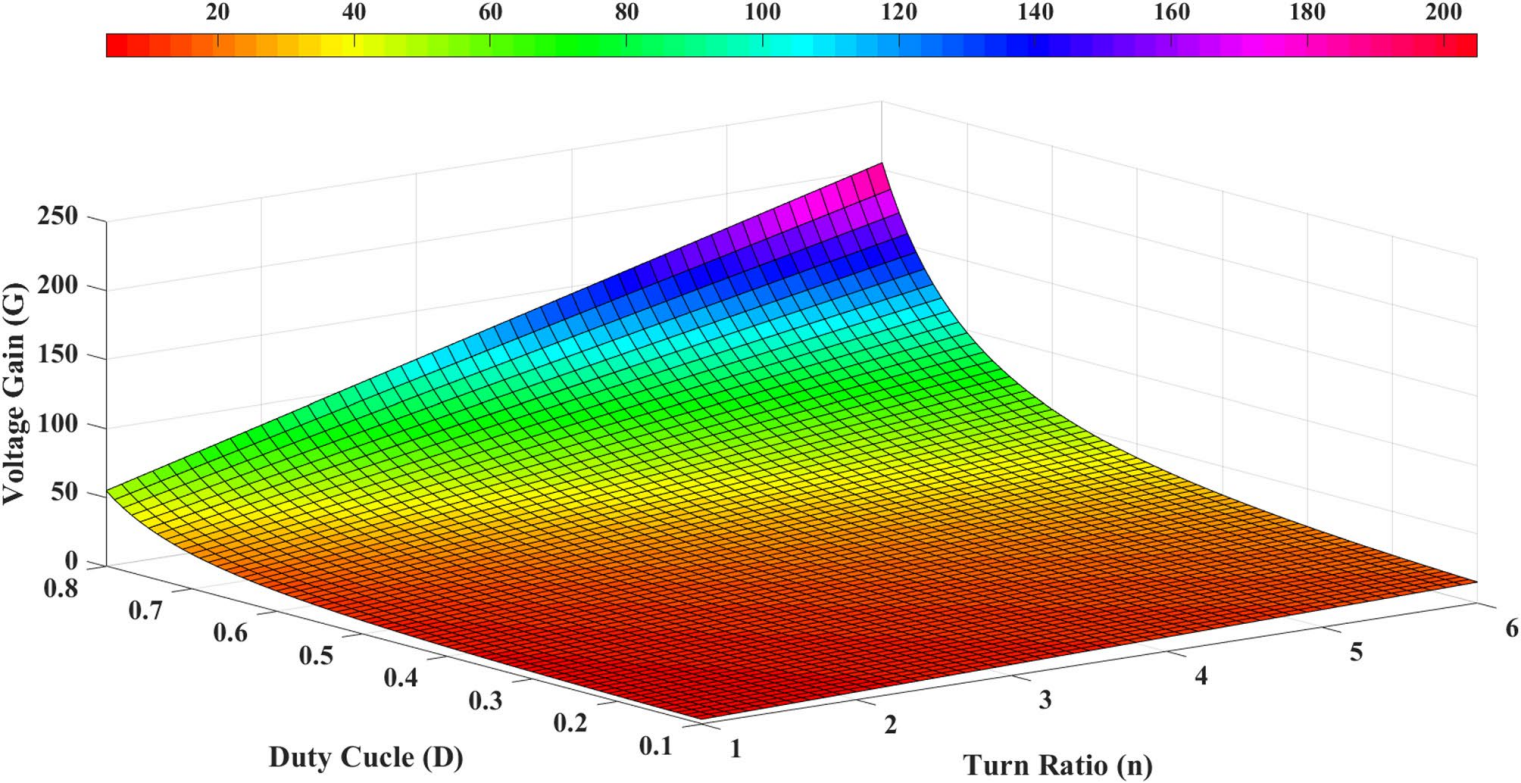
A three-dimensional plot depicting the relationship among voltage gain, n, and duty cycle D.

A three-dimensional plot of the voltage gain is illustrated in Fig. 5. This figure indicates that the proposed topology is capable of achieving extremely high voltage gains, exhibiting a quadratic dependence when suitable values of D and n are chosen.

### Voltage stresses of semiconductors

The voltage stresses experienced by the switches and diodes in the proposed circuit can be expressed as follows:

36 $${V_{S1}}=\frac{{{V_{in}}}}{{1 - D}}$$

37 $${V_{S2}}=\frac{{{V_{in}}}}{{{{(1 - D)}^2}}}$$

38 $${V_{D1}}=\frac{{{V_{in}}}}{{1 - D}}$$

39 $${V_{D2}}=\frac{{(kn+1){V_{in}}}}{{{{(1 - D)}^2}}}$$

40 $${V_{D3}}={V_{D4}}=\frac{{kn{V_{in}}}}{{{{(1 - D)}^2}}}$$

### Current analysis of the devices

The semiconductor currents during the switching subintervals can be formulated as follows:

41 $${I_{S1 - Mode1}}={I_{D1 - Mode2}}=\frac{{{I_O}(2kn - Dkn+1)}}{{{{(1 - D)}^2}}}$$

42 $${I_{S2 - Mode1}}=\frac{{{I_O}(D+2n - Dn)}}{{D(1 - D)}}$$

43 $${I_{D2 - Mode2}}=\frac{{{I_O}}}{{1 - D}}$$

44 $${I_{D3 - Mode1}}={I_{D4 - Mode1}}=\frac{{{I_O}}}{D}$$

The mathematical expressions for determining the currents in inductor L, magnetizing inductance L_m_​, and the primary N_P_ and secondary N_S_ windings are given as follows:

45 $${I_{in}}={I_L}=\frac{{{I_O}(2kn - Dkn+1)}}{{{{(1 - D)}^2}}}$$

46 $${I_{Lm}}=\frac{{{I_O}(1+n)}}{{1 - D}}$$

47 $${I_{Np - Mode1}}=\frac{{2{I_O}n}}{D}$$

48 $${I_{Np - Mode2}}=\frac{{ - {I_O}n}}{{1 - D}}$$

49 $${I_{Ns - Mode1}}=\frac{{2{I_O}}}{D}$$

50 $${I_{Ns - Mode2}}=\frac{{ - {I_O}}}{{1 - D}}$$

Based on the equivalent circuits of the operating states and applying the ampere-second balance principle, the capacitor current equations can be expressed as follows:

51 $${I_{C1 - Mode1}}= - \frac{{{I_O}(n+1)}}{{1 - D}} - \frac{{2{I_O}n}}{D}$$

52 $${I_{C1 - Mode2}}=\frac{{{I_O}(D - Dn+2n)}}{{{{(1 - D)}^2}}}$$

53 $${I_{C2 - Mode1}}={I_{C3 - Mode1}}=\frac{{{I_O}}}{D}$$

54 $${I_{C2 - Mode2}}={I_{C3 - Mode2}}= - \frac{{{I_O}}}{{1 - D}}$$

55 $${I_{Co - Mode1}}= - {I_O}$$

56 $${I_{Co - Mode2}}=\frac{{{I_O}D}}{{1 - D}}$$

### Average currents of semiconductors

The average current stresses of the power switch and diodes can be expressed as follows:

57 $${I_{S1,avg}}=\frac{{{I_O}(2n - Dn+1)D}}{{{{(1 - D)}^2}}}$$

58 $${I_{S2,avg}}=\frac{{{I_O}(2n - Dn+D)}}{{1 - D}}$$

59 $${I_{D1,avg}}=\frac{{{I_O}(2n - Dn+1)}}{{1 - D}}$$

60 $${I_{D2,avg}}={I_{D3,avg}}={I_{D4,avg}}={I_O}$$

An accurate assessment of power losses in electrical converters requires the calculation of the root mean square (RMS) currents for all components. The corresponding procedures for deriving the RMS currents of each element are presented in Eqs. (61)–(68) as follows:

61 $${I_{S1,RMS}}=\frac{{{I_O}(2n - Dn+1)\sqrt D }}{{{{(1 - D)}^2}}}$$

62 $${I_{S2,RMS}}=\frac{{{I_O}(2n - Dn+D)}}{{\sqrt D {{(1 - D)}^2}}}$$

63 $${I_{D1,RMS}}=\frac{{{I_O}(2n - Dn+1)}}{{\sqrt {{{(1 - D)}^3}} }}$$

64 $${I_{D2,RMS}}=\frac{{{I_O}}}{{\sqrt {(1 - D)} }}$$

65 $${I_{D3,RMS}}={I_{D4,RMS}}=\frac{{{I_O}}}{{\sqrt D }}$$

66 $${I_{C1,RMS}}=\left( {\frac{{{I_O}(n+1)}}{{1 - D}}+\frac{{2{I_O}n}}{D}} \right)\sqrt D +\frac{{{I_O}(D+2n - Dn)}}{{\sqrt {{{(1 - D)}^3}} }}$$

67 $${I_{C2,RMS}}={I_{C3,RMS}}=\frac{{{I_O}}}{{\sqrt D }}+\frac{{{I_O}}}{{\sqrt {(1 - D)} }}$$

68 $${I_{Co,RMS}}={I_o}\sqrt D +\frac{{{I_O}D}}{{\sqrt {1 - D} }}$$

## Boundary condition

To maintain continuous conduction mode in the proffered configuration, the minimum current of inductor L​must remain greater than zero. The minimum currents of L and ${\Delta _{iL}}$ can be expressed as:

69 $$\left\{ \begin{gathered} {V_L}={V_{in}},\frac{{Ld{I_L}}}{{dt}}={V_{in}} \Rightarrow d{I_L}=\frac{{{V_{in}}dt}}{L} \hfill \\ dt=D{T_S},{T_S}=\frac{1}{{{f_S}}},\Delta {I_L}=d{I_L},\Delta {I_L}=\frac{{{V_{in}}D}}{{L{f_S}}} \hfill \\ \end{gathered} \right.$$

70 $$\left\{ \begin{gathered} {I_{L,\hbox{min} }}={I_L} - \frac{{\Delta {I_L}}}{2} \hfill \\ {I_{L,\hbox{max} }}={I_L}+\frac{{\Delta {I_L}}}{2} \hfill \\ \end{gathered} \right.$$

The minimum inductance values of L required to sustain CCM are given as:

71 $$\left\{ \begin{gathered} {I_{L,\hbox{min} }}=0 \Rightarrow {I_L} \geqslant \frac{{\Delta {I_L}}}{2} \hfill \\ \Rightarrow \frac{{{I_O}(2kn - Dkn+1)}}{{{{(1 - D)}^2}}} \geqslant \frac{{{V_{in}}D}}{{2L{f_S}}} \hfill \\ L \geqslant \frac{{D{{(1 - D)}^4}R}}{{2{{(2kn - Dkn+1)}^2}{f_s}}} \hfill \\ \end{gathered} \right.$$

To ensure proper operation, the minimum current through capacitor CI must remain greater than zero. The minimum currents of CI​ and ${\Delta _{iLm}}$ can be defined as:

72 $$\left\{ \begin{gathered} {V_{Lm}}=\frac{{{V_{in}}k}}{{1 - D}},\frac{{{L_m}d{I_{Lm}}}}{{dt}}=\frac{{{V_{in}}}}{{1 - D}} \Rightarrow d{I_{Lm}}=\frac{{{V_{in}}dt}}{{(1 - D){L_m}}} \hfill \\ dt=D{T_S},{T_S}=\frac{1}{{{f_S}}},\Delta {I_{Lm}}=d{I_{Lm}},\Delta {I_{Lm}}=\frac{{{V_{in}}Dk}}{{(1 - D){L_m}{f_S}}} \hfill \\ \end{gathered} \right.$$

73 $$\left\{ \begin{gathered} {I_{Lm,\hbox{min} }}={I_{Lm}} - \frac{{\Delta {I_{Lm}}}}{2} \hfill \\ {I_{Lm,\hbox{max} }}={I_{Lm}}+\frac{{\Delta {I_{Lm}}}}{2} \hfill \\ \end{gathered} \right.$$

The minimum inductance of L_m_​ required to maintain continuous conduction mode is given as:

74 $$\left\{ \begin{gathered} {I_{Lm,\hbox{min} }}=0 \Rightarrow {I_{Lm}} \geqslant \frac{{\Delta {I_{Lm}}}}{2} \hfill \\ \Rightarrow \frac{{{I_O}(n+1)}}{{(1 - D)}} \geqslant \frac{{{V_{in}}Dk}}{{2(1 - D){L_m}{f_S}}} \hfill \\ {L_m} \geqslant \frac{{kDR{{(1 - D)}^2}}}{{2(n+1)(2kn - Dkn+1){f_s}}} \hfill \\ \end{gathered} \right.$$

**Fig. 6:**
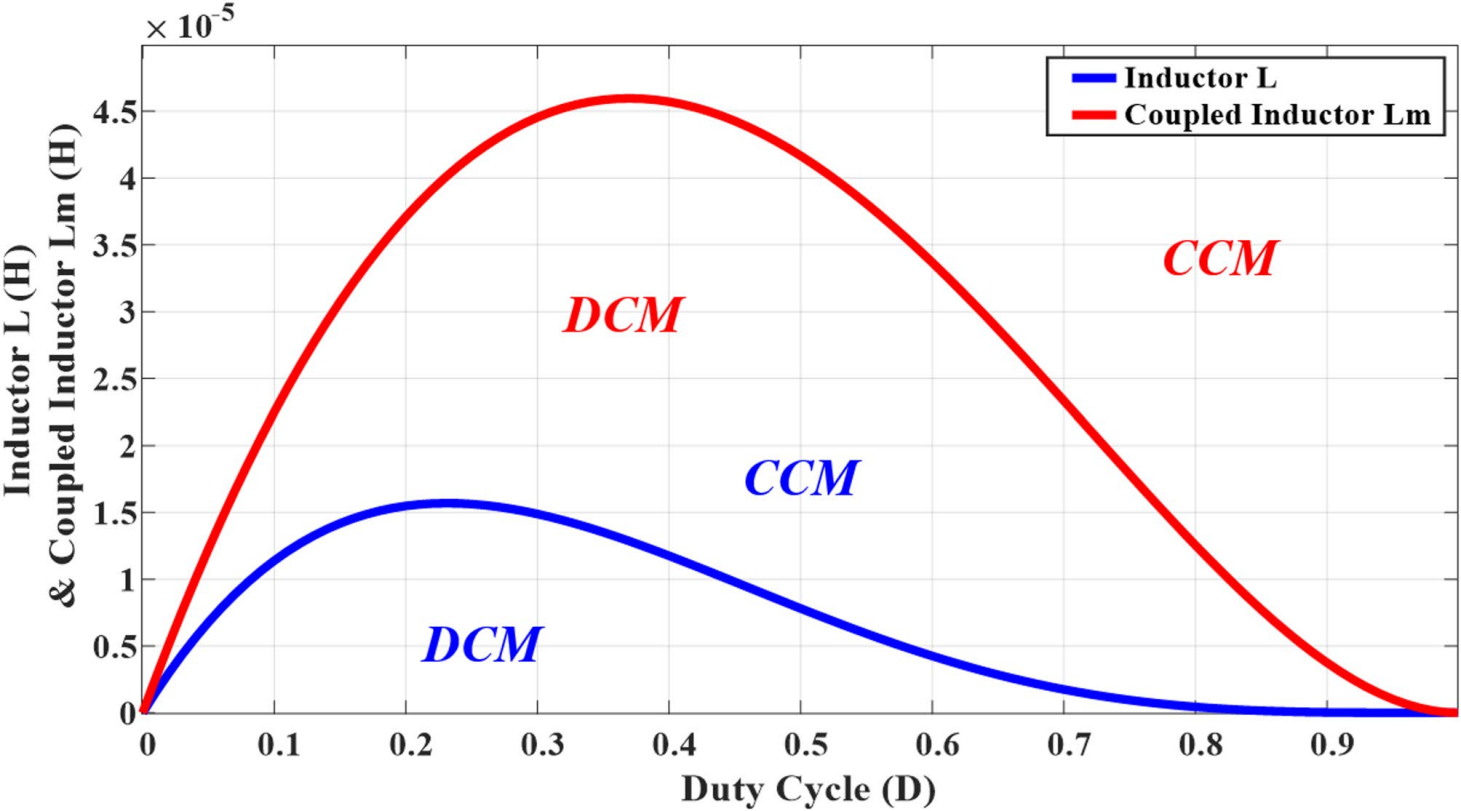
Minimum required values of both inductor L and the coupled inductor.

As illustrated in Fig. 6, the L_min_ (D) curve of the magnetizing inductor L_m_​ is positioned above that of the input inductor L. This indicates that, for identical input voltage, switching frequency, and load conditions, the magnetizing branch demands a higher inductance to remain in CCM. As a result, with the component values implemented in this work, L_m_​ shifts into discontinuous conduction mode (DCM) earlier than L, which is clearly evident from Fig. 6.

## Efficiency calculation

In this part of the study, the power dissipation due to conduction and switching is evaluated for the designed configuration with the aim of enhancing overall efficiency. The analysis incorporates key loss contributing parameters, including the intrinsic resistances of diodes (r_D_), semiconductor switches (r_S_), magnetic components such as inductors (r_Lm_), and capacitors (r_C_). Additionally, the forward voltage drops across diodes (V_FD_) and switches (V_FS_) are factored into the estimation of total losses. Based on these considerations, the conduction losses associated with both the switches and diodes are formulated as follows:

75 $${P_{Cond,{S_1}}}=\frac{{D{r_{{S_1}}}{\mkern 1mu} {{\mathrm{I}}_O}^{2}{{\left( {2n - Dn+1} \right)}^2}}}{{{{\left( {D - 1} \right)}^4}}}+\frac{{D{\mkern 1mu} {{\mathrm{V}}_{{\mathrm{F}}{{\mathrm{S}}_1}}}{\mkern 1mu} {{\mathrm{I}}_O}\left( {2n - Dn+1} \right)}}{{{{\left( {D - 1} \right)}^2}}}$$

76 $${P_{Cond,{S_1}}}=\frac{{D{r_{{S_2}}}{\mkern 1mu} {{\mathrm{I}}_O}^{2}{{\left( {2n - Dn+D} \right)}^2}}}{{{{\left( {D - 1} \right)}^2}}}+\frac{{{{\mathrm{V}}_{{\mathrm{F}}{{\mathrm{S}}_2}}}{\mkern 1mu} {{\mathrm{I}}_O}\left( {2n - Dn+D} \right)}}{{1 - D}}$$

77 $$\left\{ \begin{gathered} {P_{Cond,{D_1}}}=\frac{{{r_{{D_1}}}{\mkern 1mu} {{\mathrm{I}}_O}^{2}{{\left( {2n - Dn+1} \right)}^2}}}{{1 - D}} \hfill \\ +\frac{{{\mkern 1mu} {{\mathrm{V}}_{{\mathrm{F}}{{\mathrm{D}}_{\mathrm{1}}}}}{\mkern 1mu} {{\mathrm{I}}_O}(2n - Dn+1)}}{{1 - D}} \hfill \\ \end{gathered} \right.$$

78 $${P_{Cond,{D_2}}}={{\mathrm{V}}_{{\mathrm{F}}{{\mathrm{D}}_2}}}{{\mathrm{I}}_O}+\frac{{{{\mathrm{I}}_O}{\mkern 1mu} {{\mathrm{r}}_{{{\mathrm{D}}_2}}}}}{{1 - D}}$$

79 $${P_{Cond,{D_3}}}={{\mathrm{V}}_{{\mathrm{F}}{{\mathrm{D}}_3}}}{{\mathrm{I}}_O}+\frac{{{{\mathrm{I}}_O}^{2}{\mkern 1mu} {{\mathrm{r}}_{{{\mathrm{D}}_3}}}}}{D}$$

80 $${P_{Cond,{D_4}}}={{\mathrm{V}}_{{\mathrm{F}}{{\mathrm{D}}_4}}}{{\mathrm{I}}_O}+\frac{{{{\mathrm{I}}_O}^{2}{\mkern 1mu} {{\mathrm{r}}_{{{\mathrm{D}}_4}}}}}{D}$$

The determination of switching losses for the power devices, including switches and diodes, is conducted according to the following procedure:

81 $${P_{SW,{S_1}}}=\frac{{{{\mathrm{I}}_O}{\mkern 1mu} \times {{\mathrm{V}}_{{\mathrm{in}}}} \times {f_s} \times ({t_{off}}+{t_{on}}) \times (1+2n - Dn)}}{{6 \times {{(1 - D)}^3}}}$$

82 $${P_{SW,{S_2}}}=\frac{{{\mathrm{D}} \times {{\mathrm{I}}_O}{\mkern 1mu} \times {{\mathrm{V}}_{{\mathrm{in}}}} \times {f_s} \times ({t_{off}}+{t_{on}}) \times (D+2n - Dn)}}{{6 \times {{(1 - D)}^3}}}$$

83 $${P_{SW,{D_1}}}=\frac{{{I_{rr}} \times {V_{in}} \times {f_s} \times {t_b}}}{{6 \times (1 - D)}}$$

84 $${P_{SW,{D_2}}}=\frac{{(n+1) \times {I_{rr}} \times {V_{in}} \times {f_s} \times {t_b}}}{{6 \times {{(1 - D)}^2}}}$$

85 $${P_{SW,{D_3}}}=\frac{{n \times {I_{rr}} \times {V_{in}} \times {f_s} \times {t_b}}}{{6 \times {{(1 - D)}^2}}}$$

86 $${P_{SW,{D_4}}}=\frac{{n \times {I_{rr}} \times {V_{in}} \times {f_s} \times {t_b}}}{{6 \times {{(1 - D)}^2}}}$$

An aggregate representation of power dissipation in switches and diodes, accounting for conduction as well as switching losses, is given by:

87 $${P_{S,Tot}}={P_{Cond,{S_1}}}+{P_{SW,{S_1}}}+{P_{Cond,{S_2}}}+{P_{SW,{S_2}}}$$

88 $${P_{D,Tot}}={P_{Cond,{D_{1,2,3,4}}}}+{P_{SW,{D_{1,2,3,4}}}}$$

The conduction losses associated with capacitors C_1_, C_2_, C_3_, and C_O_ are determined as follows:

89 $${P_{Cond,{C_1}}}={r_{{C_1}}}{\left( {\sqrt D \times \left( {\frac{{{{\mathrm{I}}_O}(n+1)}}{{D - 1}} - \frac{{{{\mathrm{I}}_O}n}}{D}} \right) - \frac{{{{\mathrm{I}}_O}(D+n - Dn)}}{{\sqrt {{{(1 - D)}^3}} }}} \right)^2}$$

90 $${P_{Cond,{C_2}}}={r_{{C_2}}}{\left( {\frac{{{{\mathrm{I}}_O}}}{{\sqrt {1 - D} }}+\frac{{{{\mathrm{I}}_O}}}{{\sqrt D }}} \right)^2}$$

91 $${P_{Cond,{C_3}}}={r_{{C_3}}}{\left( {\frac{{{{\mathrm{I}}_O}}}{{\sqrt {1 - D} }}+\frac{{{{\mathrm{I}}_O}}}{{\sqrt D }}} \right)^2}$$

92 $${P_{Cond,{C_O}}}={r_{{C_O}}}{\left( {\sqrt D {{\mathrm{I}}_O}+\frac{{{\mathrm{D}}{{\mathrm{I}}_O}}}{{\sqrt {1 - D} }}} \right)^2}$$

The total energy dissipation associated with the capacitors can be formulated as follows:

93 $${P_{C,Tot}}={P_{Cond,{C_1}}}+{P_{Cond,{C_2}}}+{P_{Cond,{C_3}}}+{P_{Cond,{C_O}}}$$

The expressions used to evaluate the conduction losses of the magnetizing inductance L_m_​ and the inductor L are given below:

94 $${P_{Cond,Lm}}={r_{Lm}}I_{{Lm}}^{2}=\frac{{{\mathrm{I}}{{\mathrm{o}}^2}{r_{Lm}}{{(n+1)}^2}}}{{{{(1 - D)}^2}}}$$

95 $${P_{Cond,L}}={r_L}I_{L}^{2}=\frac{{{\mathrm{I}}{{\mathrm{o}}^2}{r_L}{{(2n - Dn+1)}^2}}}{{{{(1 - D)}^4}}}$$

The losses attributed to the inductors can be expressed through the following relations:

96 $${P_C}=kf_{s}^{\alpha }B_{m}^{\beta }$$

The core loss of the inductors, denoted as PC, is specified in units of W/kg. The parameters α, β, and k, commonly referred to as Steinmetz coefficients are typically supplied by manufacturers for various core materials. For ferrite-based cores, the exponent α generally falls within the range of 1 to 2 (1 ≤ α ≤ 2). In accordance with Faraday’s law, the expression for this loss can be formulated as:

97 $${V_L}=N\frac{{d\varphi (t)}}{{dt}}=N{A_c}\frac{{dB(t)}}{{dt}}$$

Manufacturers typically provide the core area, A_c_, for various magnetic core designs. Using this value, the maximum flux density ΔB in the inductors can be computed as follows:

98 $$\Delta B=\frac{1}{{N{A_c}}}\int\limits_{0}^{{D{T_s}}} {{V_{in}}dt}$$

The inductor core loss is represented as P_Core_= P×M, where M denotes the mass of the magnetic core. Considering that the maximum flux density is defined as Bm = ΔB/2, the core loss can subsequently be evaluated using the following expression:

99 $${P_{Core}}=kf_{s}^{\alpha }{\left( {\frac{{\Delta B}}{2}} \right)^\beta }M$$

For the inductors, an ETD 49/25/16 core is selected. The corresponding Steinmetz parameters are specified as k = 4.124 × 10^− 5^, α = 1.72, and β = 2.76. Accordingly, the aggregate power dissipation for the high step-up topology is evaluated using the following relation:

100 $${P_{Loss}}={P_{S,Tot}}+{P_{D,Tot}}+{P_{Cond,Lm}}+{P_{Cond,L}}+{P_{Cores}}+{P_{C,Tot}}$$

The efficiency of the designed circuit (η) is calculated using the following relation:

101 $$\eta =\frac{{{P_{Out}}}}{{{P_{Out}}+{P_{Loss}}}}$$

**Fig. 7:**
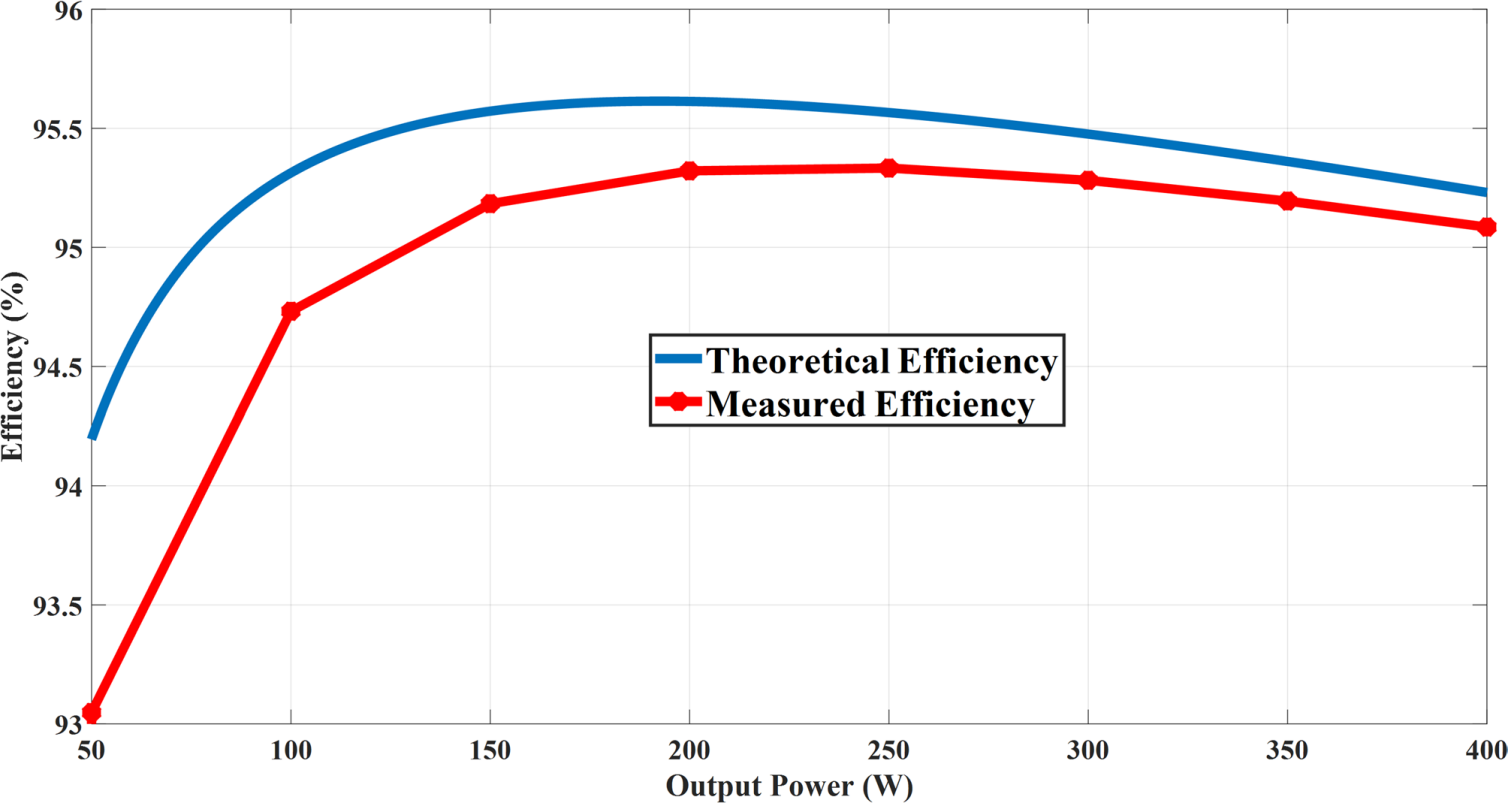
The theoretical and experimental efficiency of the proffered step-up configuration versus output power.

**Fig. 8:**
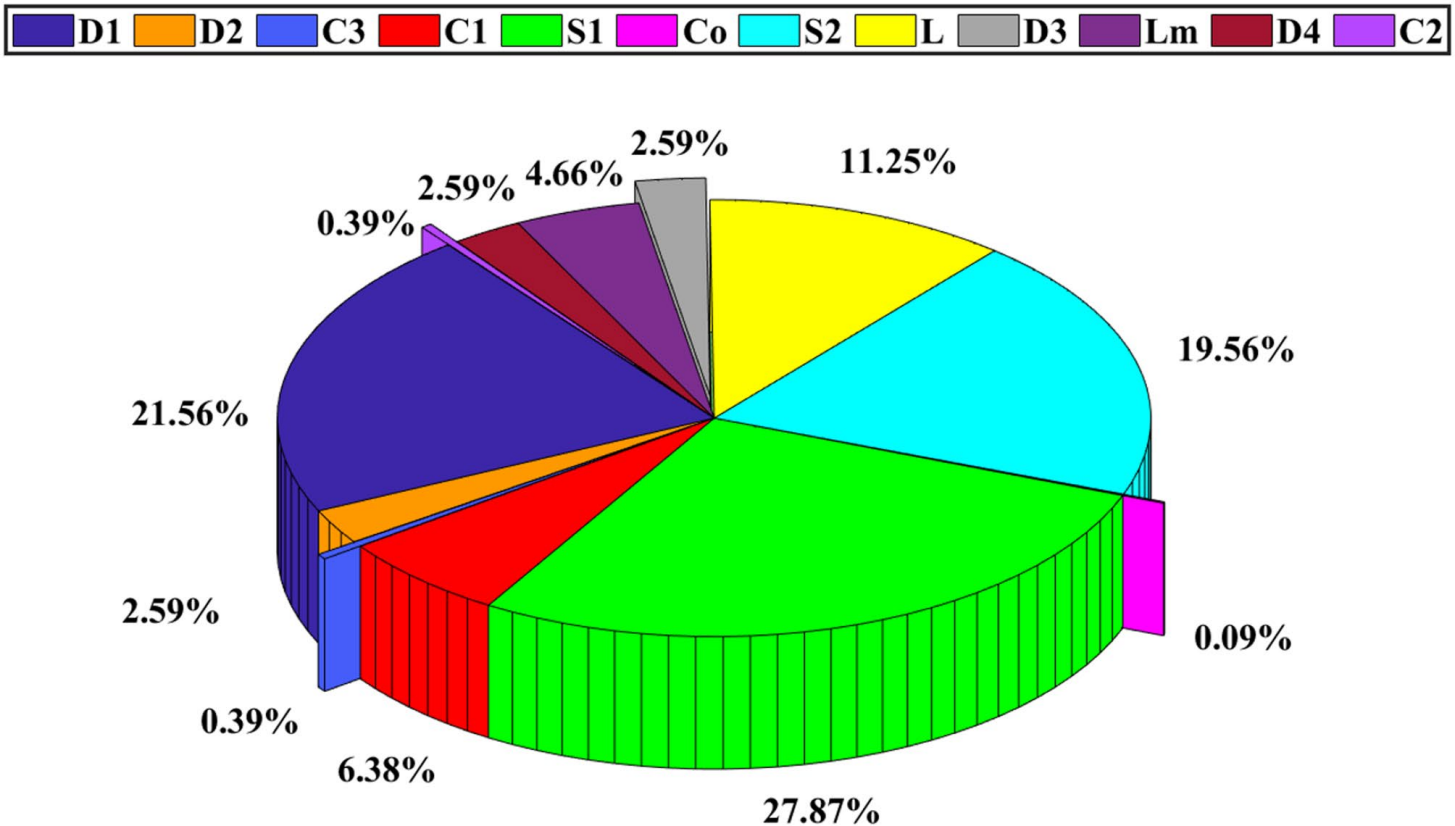
Calculated power loss percentages for the components.

**Fig. 9:**
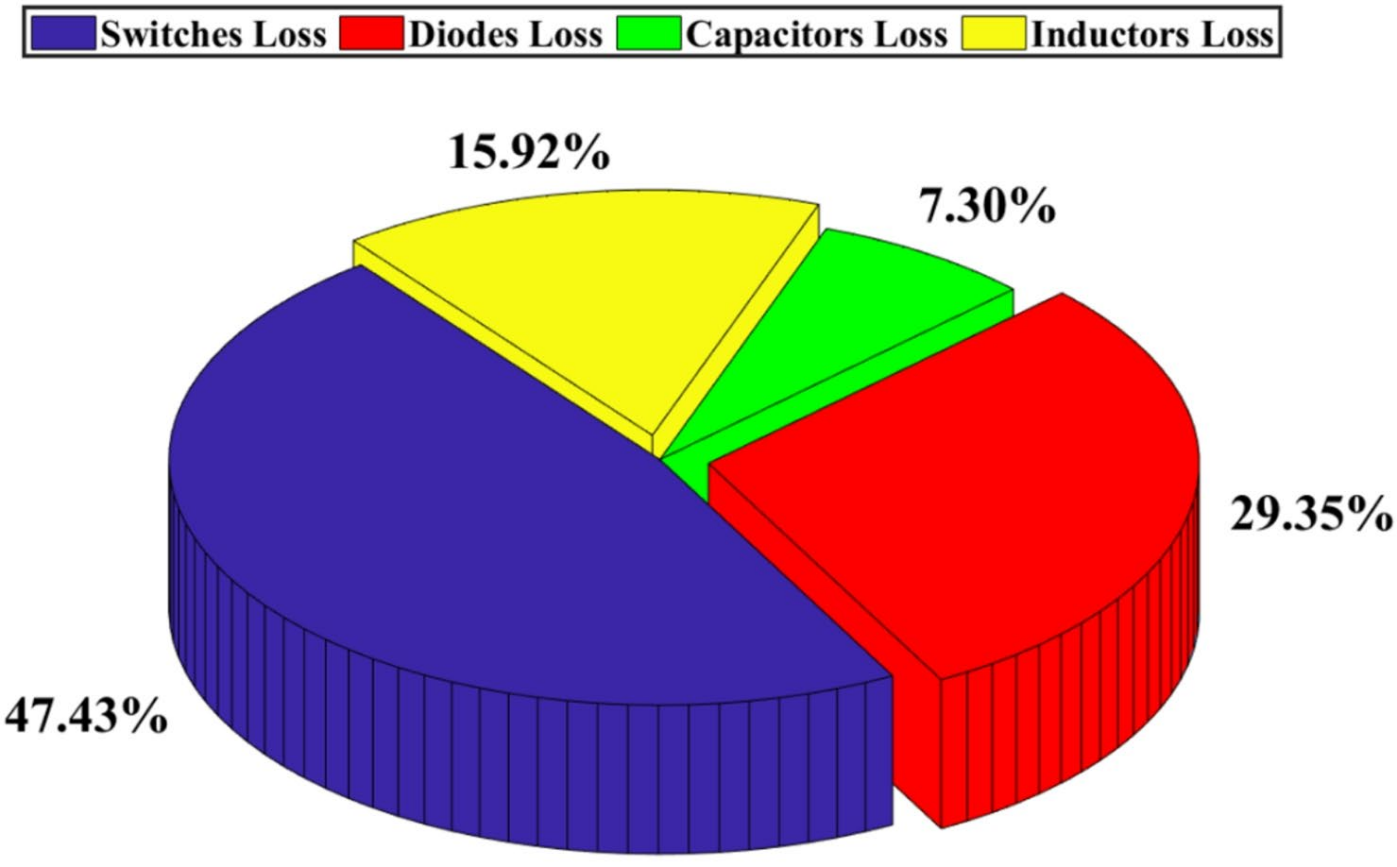
The evaluation of power dissipation percentages has been carried out for two switches, four diodes, four capacitors, and two inductors.

**Fig. 10:**
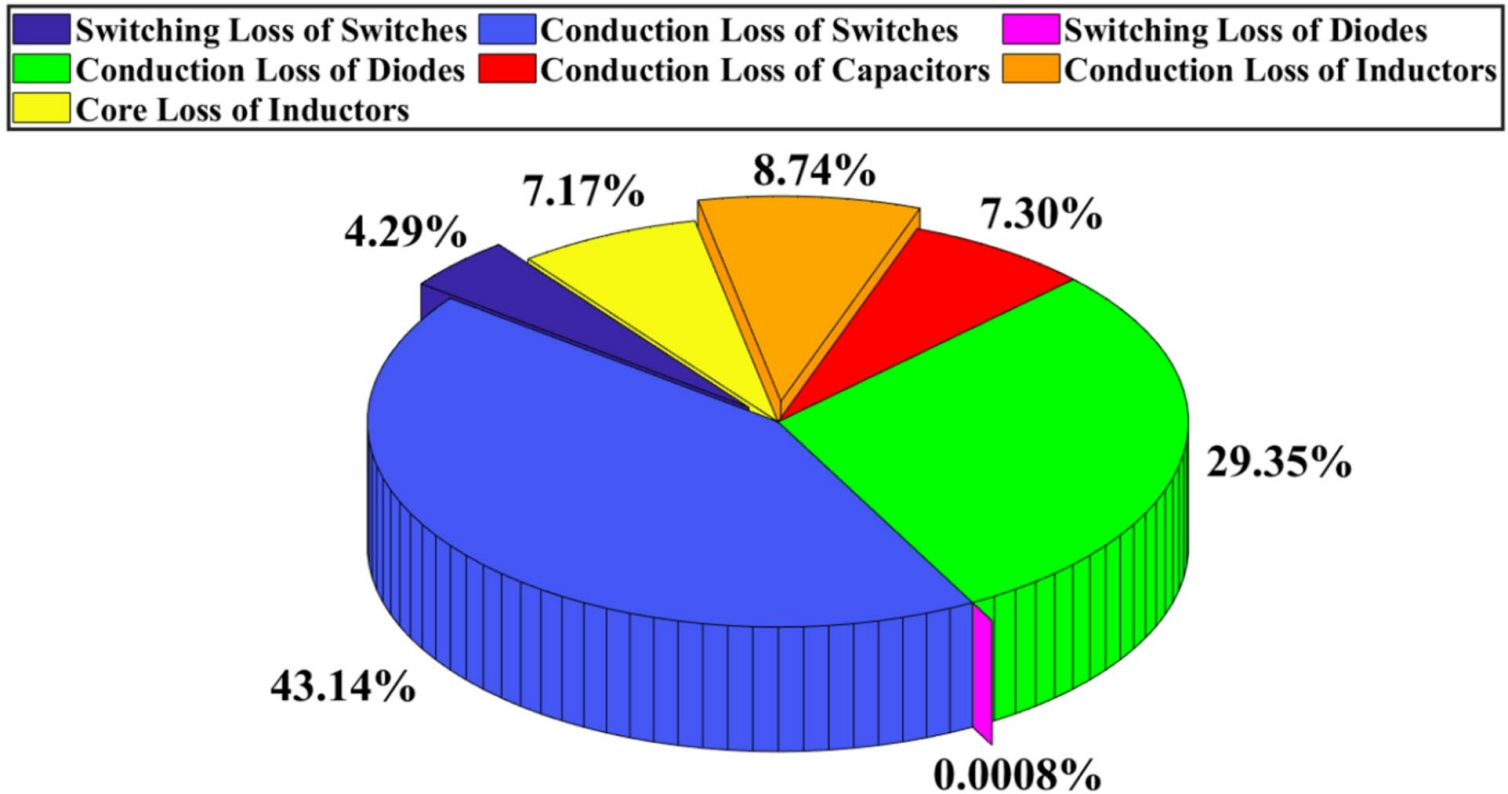
The evaluation of power dissipation reveals contributions from conduction losses (in switches, diodes, capacitors, and inductors), switching losses (in switches and diodes), as well as core losses within the inductors.

By applying equations (75) through (101), the analytical efficiency of the proposed circuit is obtained. A comparison of theoretical and experimental efficiencies versus output power is presented in Fig. 7. The breakdown of device-level power losses is illustrated in Fig. 8, where it can be observed that switch S_1_ ​contributes the highest share of losses, while capacitor C_O_​ exhibits the lowest. Figure 9 shows the calculated loss distribution across two switches, four diodes, four capacitors, and two inductors, indicating that semiconductor devices (switches and diodes) dominate the overall dissipation, accounting for approximately 76.78% of the total loss, while capacitors and inductors contribute significantly less. In Fig. 10, losses are further classified into conduction losses of switches, diodes, capacitors, and inductors; switching losses of switches and diodes; and core losses of inductors. The results demonstrate that switching-related losses are comparatively negligible when contrasted with conduction and core losses.

## Key parameter design guidance

### Capacitors design

The sizing of the capacitors is established by evaluating the average capacitor currents over all switching subintervals, while also accounting for capacitor voltages, duty cycles, the permissible fluctuation margin *x*_C_%, and a specified switching frequency of 50 kHz. On this basis, the minimum capacitance values required for C_1_​ through C_O_​ are expressed as follows:

102 $${C_1} \geq \frac{{{I_O} \times (D+2n - Dn)}}{{{f_s} \times {V_{in}} \times {x_{C1}}\% }}$$

103 $${C_2} \geq \frac{{{I_O} \times (1 - D)}}{{{f_s} \times {V_{in}} \times k \times n \times {x_{C2}}\% }}$$

104 $${C_3} \geq \frac{{{I_O} \times (1 - D)}}{{{f_s} \times {V_{in}} \times k \times n \times {x_{C3}}\% }}$$

105 $${C_O} \geq \frac{{D \times {I_O} \times {{(1 - D)}^2}}}{{{f_s} \times {V_{in}} \times (2kn - Dkn+1) \times {x_{Co}}\% }}$$

### Inductors design

The sizing criteria for inductor L and the CI are influenced by several factors, including the average current through each component, the voltage stresses across L and CI during all operating intervals, the duty ratio, the allowable current ripple limits *x*_L_% and *x*_Lm_%, as well as the selected switching frequency. Based on these parameters, the minimum inductance requirements for L and L_m_ can be formulated as:

106 $$L \geq \frac{{{V_{in}} \times D \times {{(1 - D)}^2}}}{{{f_s} \times {\mathrm{Io}}{\mkern 1mu} \times \left( {2kn - Dkn+1} \right) \times {x_L}\% }}$$

107 $${L_m} \geq \frac{{{V_{in}} \times k \times D}}{{{f_s} \times {\mathrm{Io}}{\mkern 1mu} \times \left( {{\mkern 1mu} n+1} \right) \times {x_{Lm}}\% }}$$

## Small-signal modeling

Within this section, the analysis presumes ideal operation of all power semiconductors, inductors, and capacitors. To enable accurate separation of state variables for each operating mode, parasitic effects are included by modeling inductors with series resistances r_L_​ and capacitors with series resistances r_C_​. The state-space averaging method is then employed to obtain both the averaged model and the small-signal model. This approach involves deriving system equations for every mode and averaging them across one switching period, weighted by the duration of each mode. For the two switching subintervals, the system equations are evaluated by Eq. (108), with Z = 1 and Z = 2.

108 $$\left[ {\begin{array}{*{20}{c}} {\frac{{d{i_L}}}{{dt}}} \\ {\frac{{d{i_{Lm}}}}{{dt}}} \\ {\frac{{d{v_{{C_1}}}}}{{dt}}} \\ {\frac{{d{v_{{C_2}}}}}{{dt}}} \\ {\frac{{d{v_{{C_3}}}}}{{dt}}} \\ {\frac{{d{v_{{C_O}}}}}{{dt}}} \end{array}} \right]=[{A_Z}]\left[ {\begin{array}{*{20}{c}} {{i_L}} \\ {{i_{Lm}}} \\ {{v_{{C_1}}}} \\ {{v_{{C_2}}}} \\ {{v_{{C_3}}}} \\ {{v_{{C_O}}}} \end{array}} \right]+[{B_Z}]{v_{in}}$$

The control methodology applied to the proposed converter is based on the pole-placement technique, with the small-signal representation of the circuit derived from the state-space averaged model. Within this modeling framework, the state variables and control inputs are decomposed into two parts: a steady (fixed) component $\bar {X},\bar {D}$ and a perturbation (variable) component $\tilde {x},\tilde {d}$.

109 $$\left\{ \begin{gathered} X=\bar {X}+\tilde {x} \hfill \\ D=\bar {D}+\tilde {d} \hfill \\ \end{gathered} \right.$$

Implementing the approach on the state-space averaged model, while omitting squared quantities, yields the small-signal model of the presented converter.

110 $$\left\{ \begin{gathered} \dot {\tilde {x}}=A\tilde {x}+B\tilde {u} \hfill \\ y=C\tilde {x}+D\tilde {u} \hfill \\ \end{gathered} \right.$$

The definitions of the state variables ($\tilde {x}$), control inputs ($\tilde {u}$), and output signals (y) are given below:

111 $${\tilde {x}^T}=\left[ {\begin{array}{*{20}{c}} {{{\tilde {i}}_L}}&{{{\tilde {i}}_{Lm}}}&{{{\tilde {v}}_{C1}}}&{{{\tilde {v}}_{C2}}}&{{{\tilde {v}}_{C3}}}&{{{\tilde {v}}_{CO}}} \end{array}} \right]$$

112 $$\tilde {u}=\left[ {\tilde {d}} \right]$$

113 $${y^T}=\left[ {{V_{CO}}} \right]$$

In accordance with the pole-placement method, the closed-loop poles may be assigned to any desired locations provided that the system exhibits complete state controllability. The controllability matrix of the proposed circuit is expressed as follows:

114 $${\Phi _C} = \left[ {B \vdots AB \vdots {A^{2}}B \vdots \cdots \vdots {A^{n - 1}}B} \right]$$

If the controllability matrix ${\Phi _C}$ has a rank of 6 matching the number of state variables ($\tilde {x}$) the system is deemed fully controllable. In this case, two additional integral states are introduced as:

115 $$\dot {q}(t)=r(t) - y(t)=r(t) - {\tilde {v}_{CO}}(t)$$

By incorporating the additional integral states, the system’s state-space and output equations are redefined as follows:

116 $$\begin{gathered} \left[ {\begin{array}{*{20}{c}} {\dot {\tilde {x}}(t)} \\ \cdots \\ {\dot {q}(t)} \end{array}} \right]=\left[ {\begin{array}{*{20}{c}} A& \vdots &0 \\ \cdots & \vdots & \cdots \\ { - C}& \vdots &0 \end{array}} \right]\left[ {\begin{array}{*{20}{c}} {\tilde {x}(t)} \\ \cdots \\ {q(t)} \end{array}} \right]+\left[ {\begin{array}{*{20}{c}} B \\ \cdots \\ 0 \end{array}} \right]\tilde {u}(t)+\left[ {\begin{array}{*{20}{c}} 0 \\ \cdots \\ I \end{array}} \right]r(t) \hfill \\ y(t)=\left[ {\begin{array}{*{20}{c}} C& \vdots &0 \end{array}} \right]\left[ {\begin{array}{*{20}{c}} {\tilde {x}(t)} \\ \cdots \\ {q(t)} \end{array}} \right] \hfill \\ \end{gathered}$$

Within this formulation, the term r (t) corresponds to the reference input vector and is specified as follows:

117 $$r(t)={\left[ {{V_{CO,ref}}} \right]^T}$$

In accordance with Eq. (116), the reformulated matrices $\bar {A}$ and $\bar {B}$ are defined as:

118 $$\bar {A}=\left[ {\begin{array}{*{20}{c}} A& \vdots &0 \\ \cdots & \vdots & \cdots \\ { - C}& \vdots &0 \end{array}} \right],\bar {B}=\left[ {\begin{array}{*{20}{c}} B \\ \cdots \\ 0 \end{array}} \right]$$

For the system defined by Eq. (116), the controllability matrix is expressed as:

119 $${\bar {\Phi }_C}=\left[ {\begin{array}{*{20}{c}} B& \vdots &{A{\Phi _C}} \\ \cdots & \vdots & \cdots \\ 0& \vdots &{ - C{\Phi _C}} \end{array}} \right]=\underbrace {{\left[ {\begin{array}{*{20}{c}} B& \vdots &A \\ \cdots & \vdots & \cdots \\ 0& \vdots &{ - C} \end{array}} \right]}}_{M}\left[ {\begin{array}{*{20}{c}} I& \vdots &0 \\ \cdots & \vdots & \cdots \\ 0& \vdots &{{\Phi _C}} \end{array}} \right]$$

When ${\Phi _C}$ is full-rank, the system in Eq. (116) achieves complete controllability if the rank of matrix M is equal to n + m. Here, n represents the number of state variables ($\tilde {x}$) and mmm denotes the number of outputs (y). In this case, the gain matrix K is computed as:

120 $$\tilde {u}(t)= - K\left[ {\begin{array}{*{20}{c}} {\tilde {x}(t)} \\ \cdots \\ {q(t)} \end{array}} \right]= - \left[ {\begin{array}{*{20}{c}} {{K_x}}& \vdots &{{K_q}} \end{array}} \right]\left[ {\begin{array}{*{20}{c}} {\tilde {x}(t)} \\ \cdots \\ {q(t)} \end{array}} \right]$$

The feedback gain matrices K_x_ and K_q_ are written as follows:

121 $$\begin{gathered} {K_x}=\left[ {\begin{array}{*{20}{c}} {{K_{11}}}&{{K_{12}}}&{{K_{13}}}&{{K_{14}}}&{{K_{15}}}&{{K_{16}}} \end{array}} \right] \hfill \\ {K_q}=\left[ {{{K^{\prime}}_{16}}} \right] \hfill \\ \end{gathered}$$

Substituting (120) in (116) the following equation is:

122 $$\begin{gathered} \left[ {\begin{array}{*{20}{c}} {\dot {\tilde {x}}(t)} \\ \cdots \\ {\dot {q}(t)} \end{array}} \right]=\left[ {\begin{array}{*{20}{c}} {A - B{K_x}}& \vdots &{ - B{K_q}} \\ \cdots & \vdots & \cdots \\ { - C}& \vdots &0 \end{array}} \right]\left[ {\begin{array}{*{20}{c}} {\tilde {x}(t)} \\ \cdots \\ {q(t)} \end{array}} \right]+\left[ {\begin{array}{*{20}{c}} 0 \\ \cdots \\ I \end{array}} \right]r(t) \hfill \\ y(t)=\left[ {\begin{array}{*{20}{c}} C& \vdots &0 \end{array}} \right]\left[ {\begin{array}{*{20}{c}} {\tilde {x}(t)} \\ \cdots \\ {q(t)} \end{array}} \right] \hfill \\ \end{gathered}$$

**Fig. 11:**
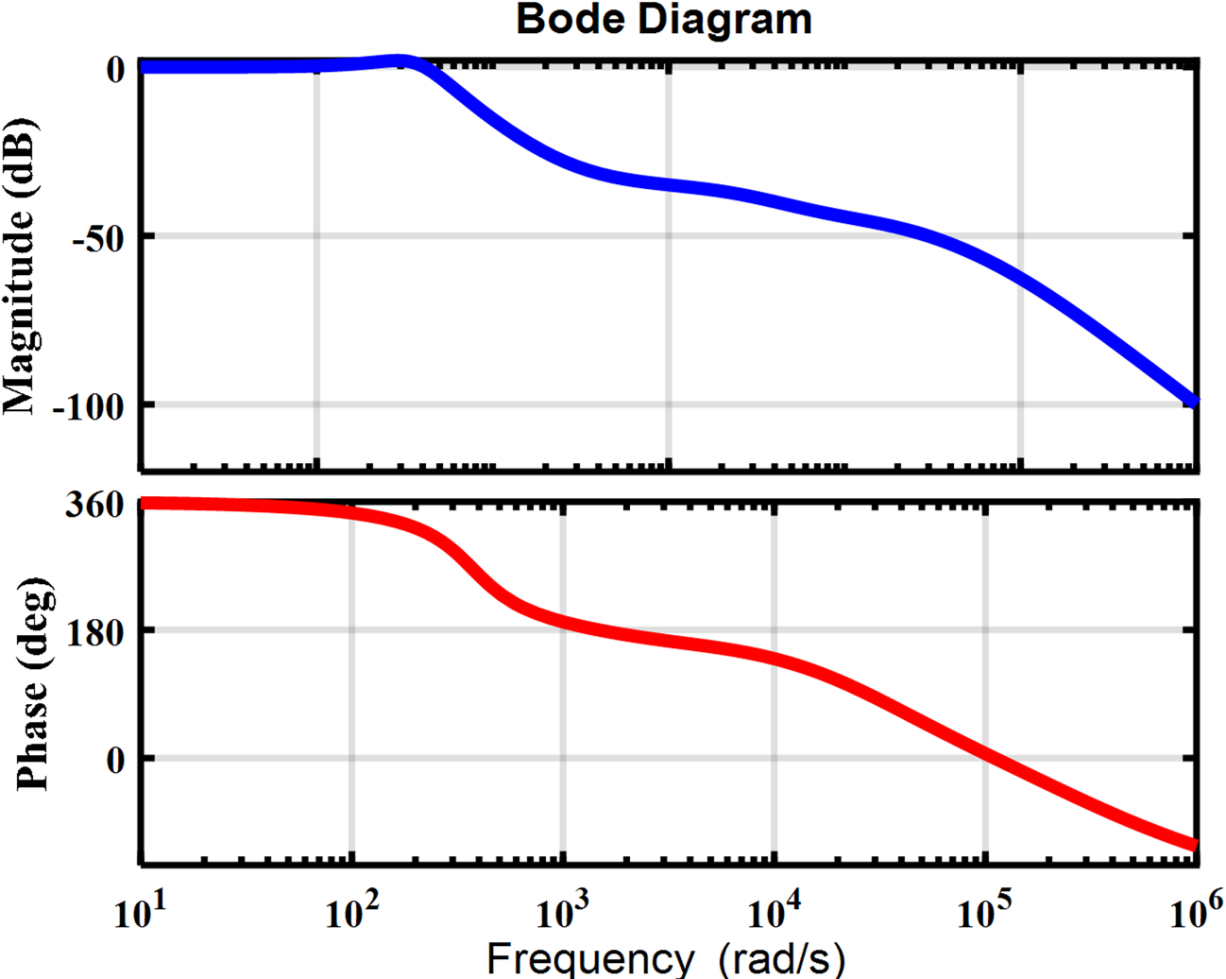
Transfer function Bode diagram corresponding to the voltage across the output capacitor.

**Fig. 12:**
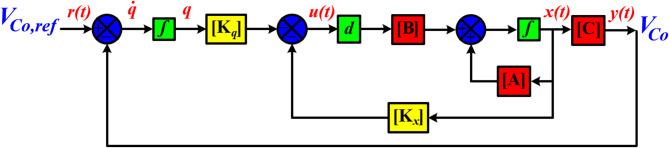
Block diagram illustrating the pole-placement control methodology.

**Fig. 13:**
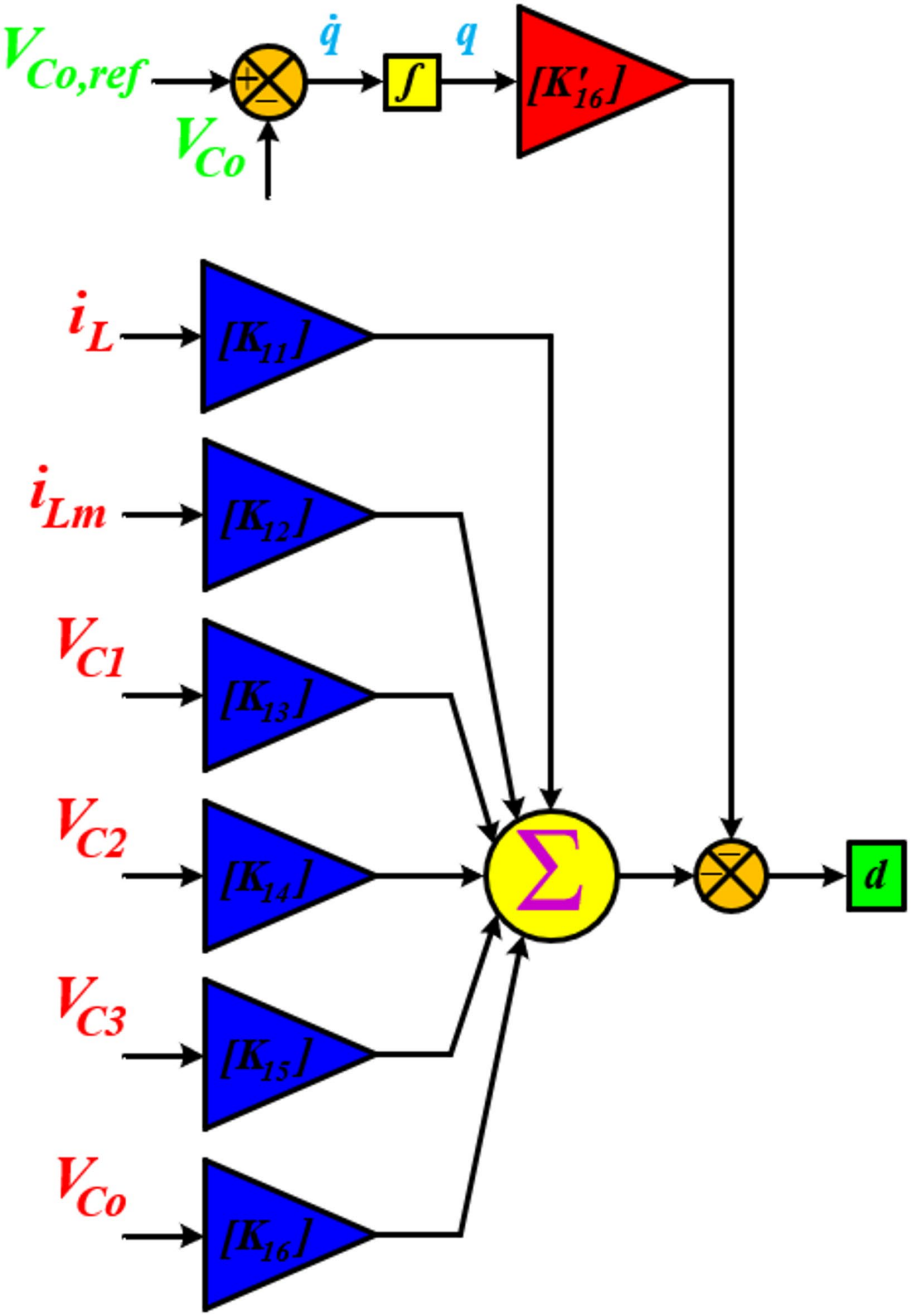
Voltage regulation loop of the output capacitor C_O_ in the designed topology.

To guarantee acceptable stability margins, the pole-placement design is carried out such that the Gain Margin (GM) is no less than 10 and the Phase Margin (PM) lies within the range of 60°–80°. A trial-and-error approach is applied to appropriately locate the closed-loop poles. Using this method, the Bode plot of the control system for the proposed converter is shown in Fig. 11. As illustrated, the gain margin for the capacitor C_O_​ satisfies the requirement with GM (V_CO_) > 10, while the phase margin of the closed-loop control path associated with V_CO_​ is calculated as 73.2731°, which falls within the acceptable range. Furthermore, Figs. 12 and 13 present the block diagram of the pole-placement control scheme and the voltage regulation loop of the output capacitor C_O_, respectively.

## Comparison study

A broad comparison was carried out to showcase the merits of the proposed converter. In total, 20 competing designs were reviewed: 4 reported in 2025, 8 in 2024, 4 in 2023, 1 in 2022, and 3 in 2021. Table 1 compiles a feature-by-feature contrast between the proposed topology and these alternatives, covering voltage gain, peak switch voltage stress, rated power, efficiency, device count, and whether a common ground is supported. The entries draw on converters cited in references^14–29^.

**Fig. 14:**
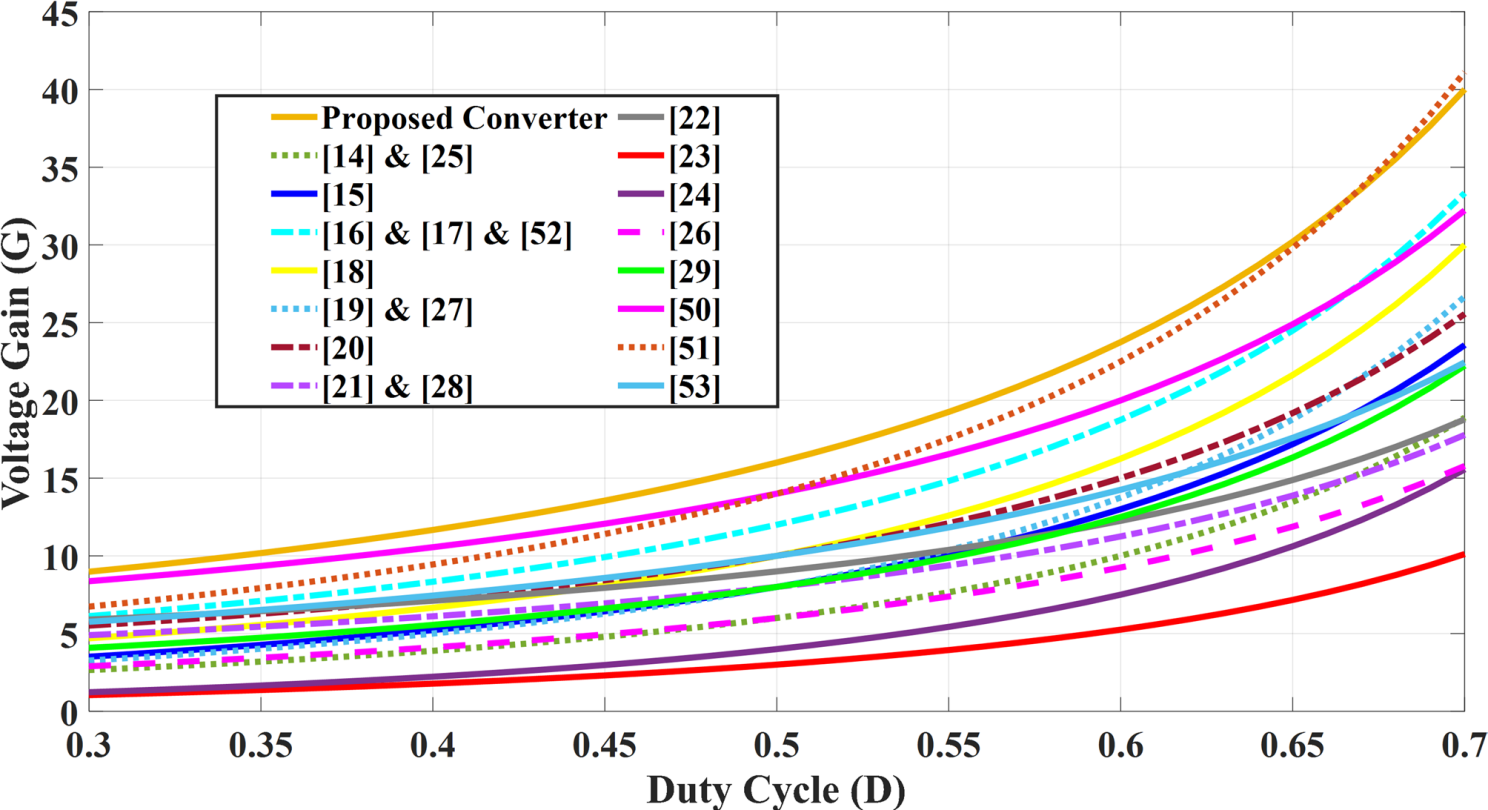
Voltage gain variations per different duty cycles for the compared step-up circuits.

Because this work targets high step-up performance, voltage gain serves as the principal evaluation metric. Using the data in Table 1; Fig. 14 plots gain versus duty cycle for all examined boost topologies. The curves indicate that the proposed configuration delivers a higher gain than any of the designs summarized in Table 1.

**Fig. 15:**
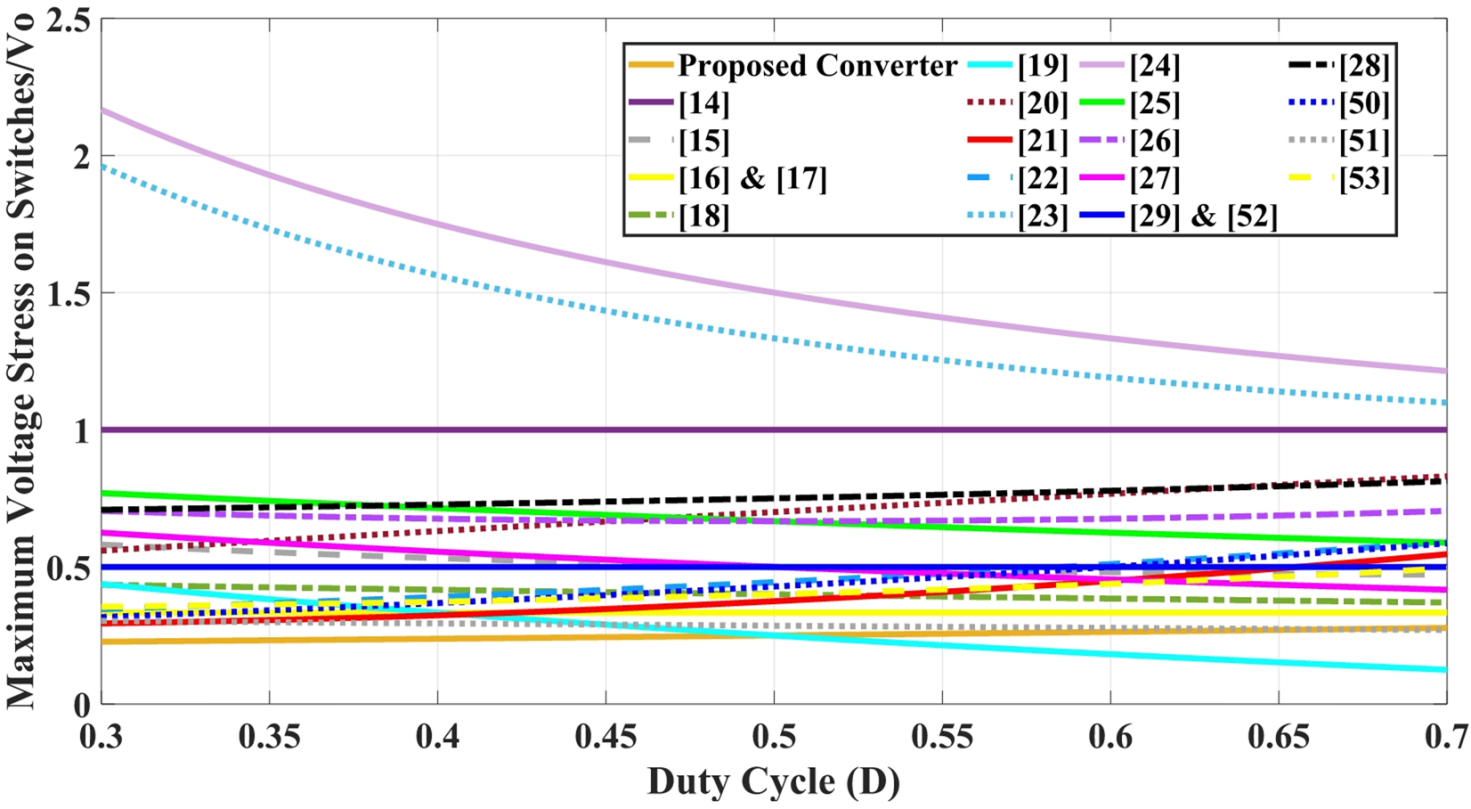
Comparison of the maximum voltage stress on switches versus duty cycle.

Switch voltage ratings appear in the second column of Table 1, and their variation with duty cycle is depicted in Fig. 15. Across most operating points, the proposed circuit exhibits excellent control of peak switch stress relative to other topologies. The one exception arises for the design in^19^: above a 50% duty cycle, its maximum switch stress falls below that of the proposed converter; below 50%, the proposed converter has the lowest stress among all compared designs. Notably, operation at duty cycles under 50% generally improves converter behavior and reduces losses, owing to the smaller duty ratio. A further advantage is that the switches in the proposed structure experience comparatively low voltage, enabling the use of a smaller inductor during design.

The third column of Table 1 summarizes performance metrics most notably rated power and efficiency. The proposed converter is designed for 400 W output and delivers 95.23% efficiency, as verified in Fig. 7. By contrast, the majority of entries in Table 1 are rated below 400 W. Even at the 400 W operating point, the proposed design sustains 95.23% efficiency, exceeding the efficiency reported for most competing converters. Many compared designs operate at lower power levels yet still exhibit lower efficiency relative to the proposed topology.

**Fig. 16:**
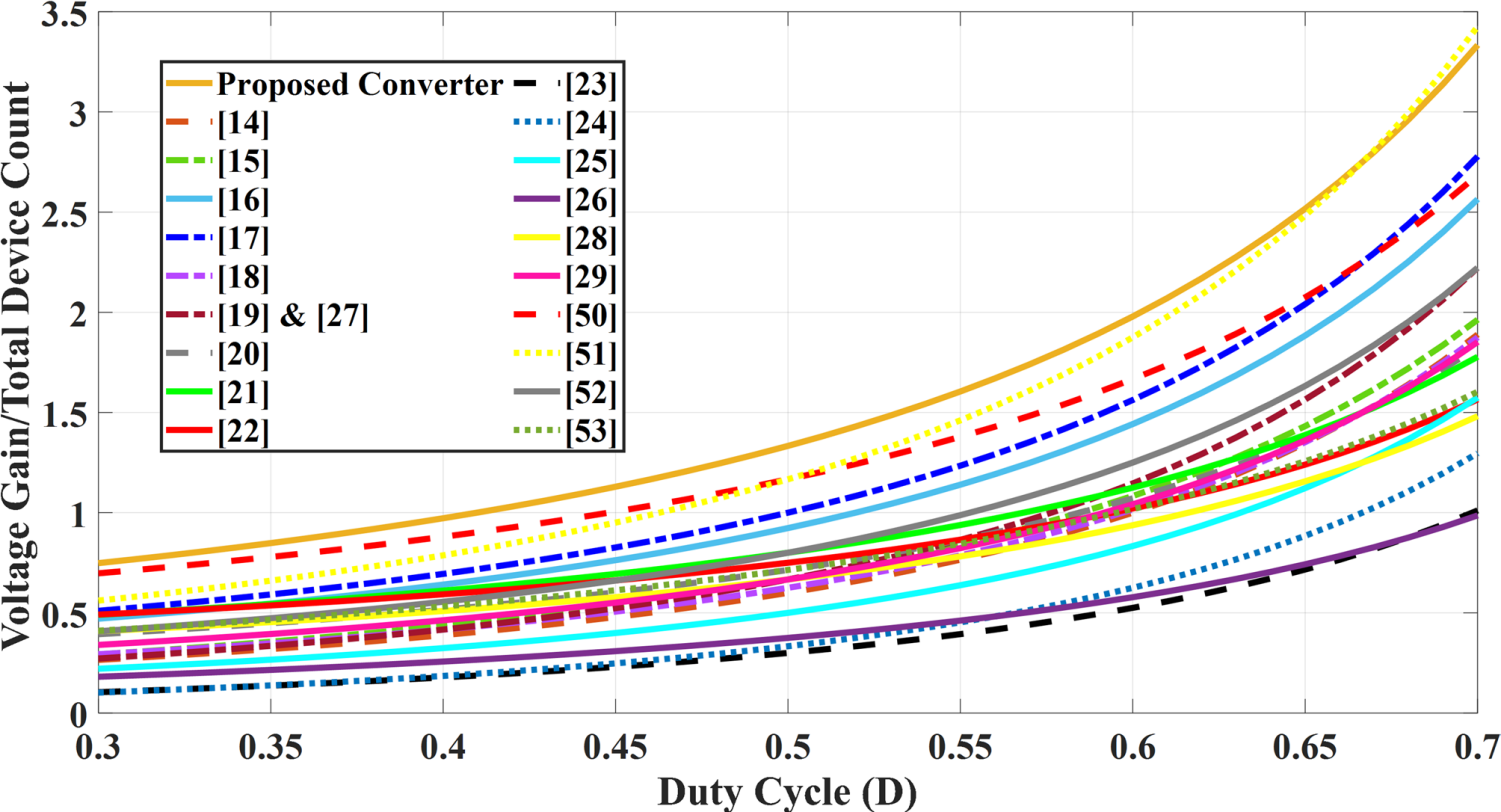
Ratio of voltage gain to total device count across different duty-cycle values.

The fourth column reports the component counts, and the final column aggregates the total device count (and related sub-tallies). A desirable architecture minimizes devices while maximizing voltage gain. To assess this trade-off, Fig. 16 plots voltage gain versus total device count across all configurations. The curves show that the proposed circuit achieves a higher gain per total device count than any of the alternatives, implying a cost advantage for equivalent performance. The last section also evaluates common-ground capability between input and output a key consideration, since topologies lacking a shared ground are typically more susceptible to electromagnetic interference, which can degrade performance and limit application scope. Unlike the converters in^14,15,21,22,24^, the proposed design provides a common ground. Table 2 presents a comparative cost analysis between the proposed converter and several alternative topologies. The estimated prices of the required components were derived from publicly available listings on online marketplaces such as Amazon and eBay, and the summarized cost data are reported in Table 2. The comparison clearly indicates that the proposed design offers the lowest overall cost among the evaluated configurations, making it the most economically efficient option. Overall, the comparative results underscore the proposed converter’s strengths: higher voltage gain, lower switch-voltage stress, superior gain per total device count across duty cycles, and high efficiency at the 400 W level.

**Table 1:** Comparison between the proffered configuration and other designs.

Ref/PY*	Voltage gain	Maximum voltage stress on switches	Power (W)/efficiency	No. of components					Total component count/common ground
				S	D	C	L	CI	
PC*	$\frac{{2kn - Dkn+1}}{{{{(1 - D)}^2}}}$	$\frac{{{V_O}}}{{2kn - Dkn+1}}$	400/95.23%	2	4	4	1	1	12/Yes
^14^ 2025	$\frac{{1+D}}{{{{(1 - D)}^2}}}$	${V_O}$	500/94.3%	2	3	3	2	0	10/No
^15^ 2025	$\frac{{(1+3D - 2{D^2})}}{{{{(1 - D)}^2}}}$	$\frac{{{V_O}}}{{1+3D - 2{D^2}}}$	100/96%	2	3	4	3	0	12/No
^16^ 2025	$\frac{{n+1}}{{{{(1 - D)}^2}}}$	$\frac{{{V_O}}}{{n+1}}$	360/95.2%	1	5	5	0	2	13/ Yes
^17^ 2025	$\frac{{n+1}}{{{{(1 - D)}^2}}}$	$\frac{{{V_O}}}{{n+1}}$	400/95%	1	4	4	2	1	12/ Yes
^18^ 2024	$\frac{{2+D}}{{{{(1 - D)}^2}}}$	$\frac{{{V_O}}}{{2+D}}$	50/87%	1	6	6	3	0	16/ Yes
^19^ 2024	$\frac{{2D+n - 1}}{{{{(1 - D)}^2}}}$	$\frac{{(1 - D){V_o}}}{{2D+n - 1}}$	210/93.4%	2	4	4	1	1	12/Yes
^20^ 2024	$\frac{{3 - D}}{{{{(1 - D)}^2}}}$	$\frac{{(1+2D - {D^2}){V_o}}}{{3 - D}}$	300/94.5%	1	6	4	3	0	14/Yes
^21^ 2024	$\frac{{3 - 2D}}{{{{(1 - D)}^2}}}$	$\frac{{(1 - 2D{{(1 - D)}^2}){V_o}}}{{3 - 2D}}$	300/93.6%	2	3	3	2	0	10/No
^22^ 2024	$\frac{{{{(2 - D)}^2}}}{{{{(1 - D)}^2}}}$	$\frac{{{V_o}}}{{{{(2 - D)}^2}}}$	400/93.3%	2	4	4	2	0	12/No
^23^ 2024	$\frac{{D(2 - D)}}{{{{(1 - D)}^2}}}$	$\frac{{{V_O}}}{{D(2 - D)}}$	72/95%	2	2	3	3	0	10/ Yes
^24^ 2024	$\frac{{2D}}{{{{(1 - D)}^2}}}$	$\frac{{(1+D){V_O}}}{{2D}}$	60/97%	2	3	4	3	0	12/ No
^25^ 2024	$\frac{{1+D}}{{{{(1 - D)}^2}}}$	$\frac{{{V_O}}}{{1+D}}$	100/94%	1	4	4	3	0	12/ Yes
^26^ 2023	$\frac{{1+2D - 2{D^2}}}{{{{(1 - D)}^2}}}$	$\frac{{{V_o}}}{{1+2D - 2{D^2}}}$	200/90%	1	5	6	4	0	16/Yes
^27^ 2023	$\frac{{n - 1+nD}}{{{{(1 - D)}^2}}}$	$\frac{{(n - 1){V_o}}}{{n - 1+nD}}$	100/93%	1	4	4	2	1	12/Yes
^28^ 2023	$\frac{{3 - 2D}}{{{{(1 - D)}^2}}}$	$\frac{{(2 - D){V_o}}}{{3 - 2D}}$	400/95.1%	1	5	4	1	1	12/Yes
^29^ 2023	$\frac{2}{{{{(1 - D)}^2}}}$	$\frac{{{V_o}}}{2}$	42/93.4%	1	5	4	0	2	12/Yes
^50^ 2021	$\frac{{1+D+2n(1 - D)}}{{{{(1 - D)}^2}}}$	$\frac{{(1+D){V_o}}}{{1+D+2n(1 - D)}}$	200/94%	2	4	4	1	1	12/Yes
^51^ 2021	$\frac{{1+n+D}}{{{{(1 - D)}^2}}}$	$\frac{{{V_o}}}{{1+n+D}}$	400/94.4%	2	4	4	0	2	12/Yes
^52^ 2021	$\frac{{1+n}}{{{{(1 - D)}^2}}}$	$\frac{{{V_o}}}{2}$	200/95%	1	7	5	1	1	15/Yes
^53^ 2022	$\frac{{1+n - n{D^2}}}{{{{(1 - D)}^2}}}$	$\frac{{{V_o}}}{{1+n - n{D^2}}}$	400/96.1%	1	6	5	1	1	14/Yes

PY*: Published Year PC*: Proposed Converter.

## Experimental results

To validate the design’s operation and accuracy, a 400 W laboratory prototype was built, specified for 25 V input and 400 V output. The prototype’s full parameters are summarized in Table 3. Using a high frequency current probe (MICSIG CP2100A, 800 kHz) together with a voltage probe, we captured and analyzed current and voltage waveforms across the key devices.

**Fig. 17:**
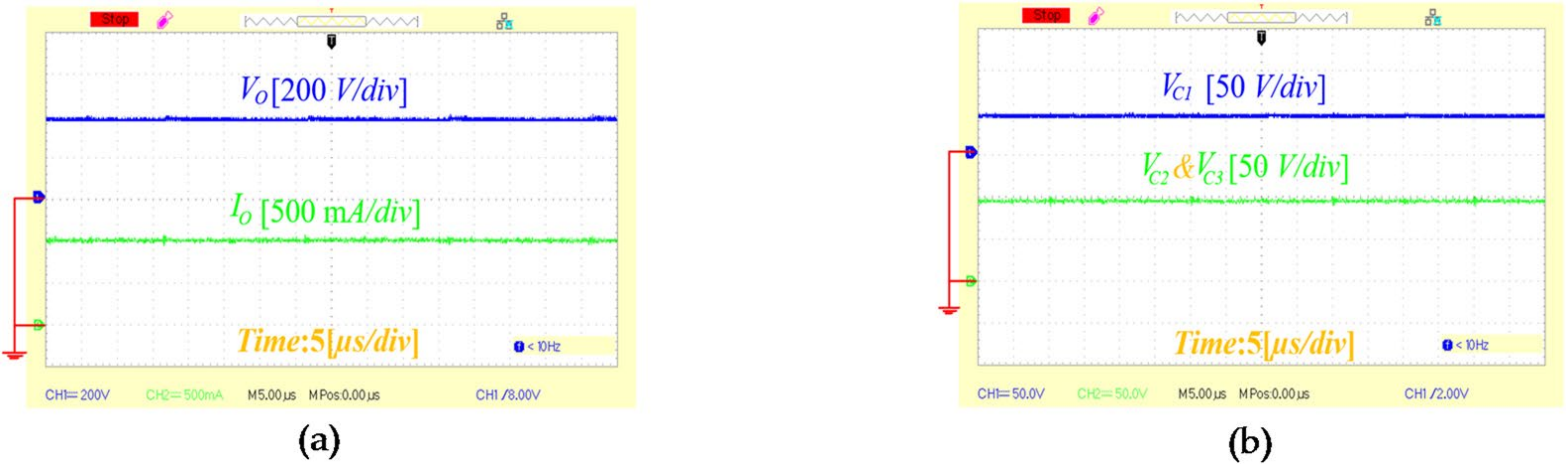
The experimental waveforms of output port and capacitors, (**a**) voltage and current of the output port, (**b**) voltage across the capacitors C_1_, C_2_ and C_3_.

Figure 17a records an output of 390 V at 1.03 A. From (34), the expected output is 400 V, which closely matches the measured 390 V. Likewise, (32) and (33) predict 50 V across C_1_ and 100 V across C_2_ and C_3_; the experiments show 48 V on C_1_ and 96 V on C_2_ and C_3_, as plotted in Fig. 17b, confirming strong agreement with theory.

**Fig. 18:**
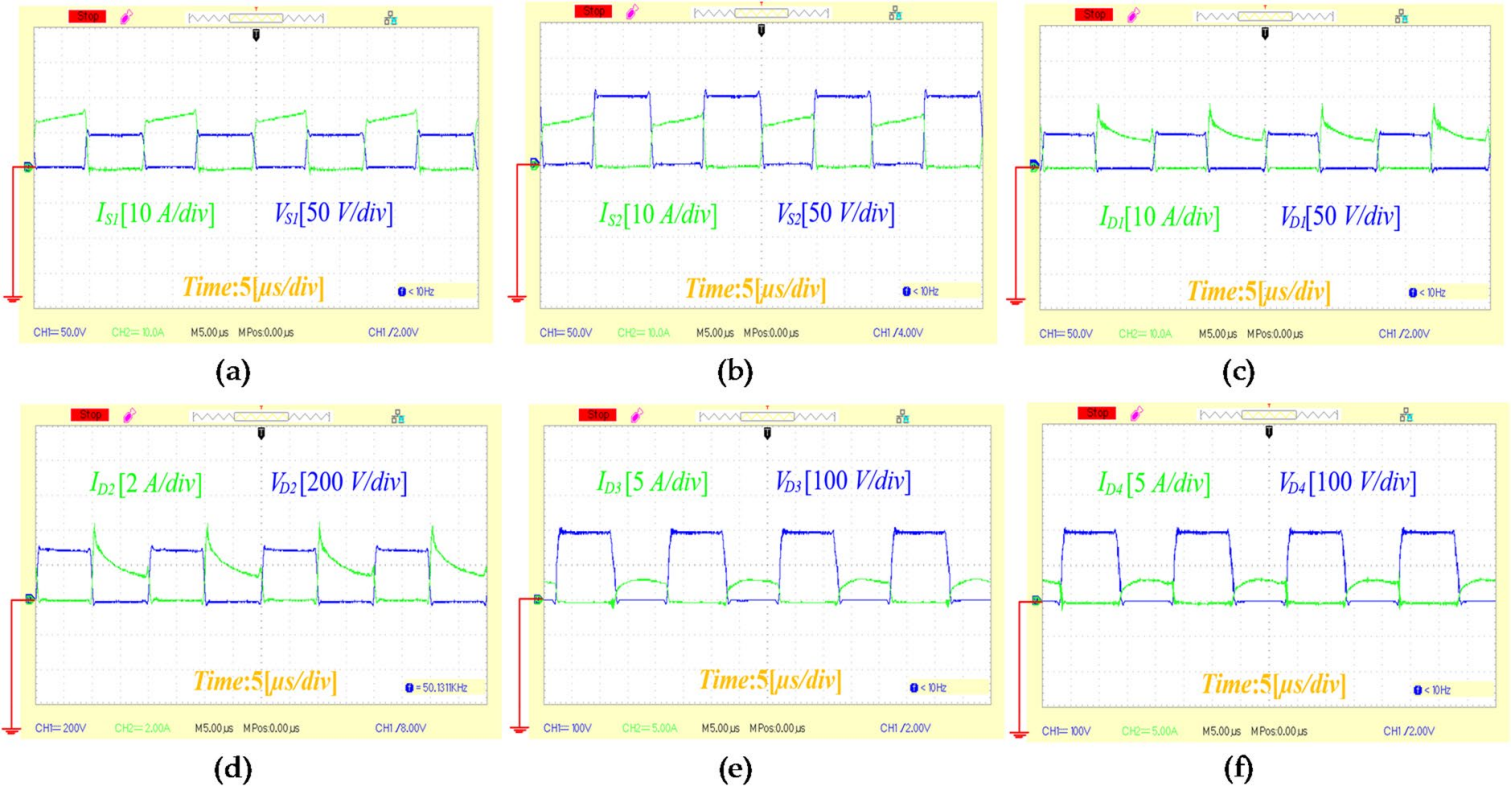
The experimental waveforms of power switches and diode, (**a**) voltage and current of the power switch S_1_, (**b**) voltage and current of the power switch S_2_, (**c**) voltage and current of the diode D_1_, (**d**) voltage and current of the diode D_2_, (**e**) voltage and current of the diode D_3_, (**f**) voltage and current of the diode D_4_.

In Fig. 18a, the waveforms of switch S_1_ show that during first mode it is subjected to only a small portion of the output voltage about 12.5% which helps limit switching losses. The first switching subinterval for the power path through S_2_ is captured experimentally in Fig. 18b. The measurements in Fig. 18a,b align closely with the analytical results derived from (36), (37), (41), and (42), indicating excellent agreement between model and experiment. Figure 18c, d present the voltage and current of diodes D_1_ and D_2_; the observed values are in strong accordance with predictions from (38), (39), (41), and (43). Similarly, the diode behaviors for D_3_ and D_4_, shown in Fig. 18e,f, are consistent with theoretical expectations based on (40) and (44), confirming tight correspondence between calculated and measured results.

**Table 2:** Cost comparison between the proposed converter and other structures.

Ref	Cost of switches	Cost of diodes	Cost of capacitors	Cost of cores	Total cost	Cost per watt
PC	2 × 1.18$	1 × 0.405$ 3 × 0.342$	1 × 0.717$ 2 × 0.799$ 1 × 1.7$	2 × 3.3$	**14.406$**	**0.036$**
^17^	1 × 1.18$	2 × 0.405$ 2 × 0.342$	2 × 0.717$ 1 × 0.799$ 1 × 1.7$	3 × 3.3$	**16.5$**	**0.041$**
^19^	2 × 4.14$	1 × 0.165$ 2 × 0.52$ 1 × 0.353$	1 × 2.2$ 1 × 1.52$ 2 × 1.61$	1 × 2.73$ 1 × 6.87$	**26.37$**	**0.125$**
^47^	1 × 1.17$	3 × 0.52$ 2 × 0.75$	2 × 1.75$ 2 × 3.67$	2 × 3.31$	**21.69$**	**0.108$**
^49^	The total cost, as computed by the authors in^49^, is equal to: **39.15$**					**0.078$**

**Table 3:** Hardware component parameters in the prototype.

Parameters	Values
Rated power (*P*_*o*_)	400 *W*
Input voltage	25 V
Output voltage	400 V
Switching frequency (*f*_*s*_)	50 kHz
Turns ratio *n* (*N*_*S*_*/N*_*P*_)	2
Magnetizing inductor (*L*_*m*_)	300 *µH*
Leakage inductor (*L*_*k*_)	3 *µH*
Power switches	IRF2807PbF
Diode (*D*_*1*_)	MBR30300CT
Diodes (*D*_*2*_,*D*_*3*_,*D*_*4*_)	Mur1560G
Cores type	ETD 49/25/16
Capacitor (*C*_*1*_)	68 µF/100V
Capacitors (*C*_*2*_, *C*_*3*_)	68 µF/200V
Capacitor (*C*_***O***_)	100 µF/450V

**Fig. 19:**
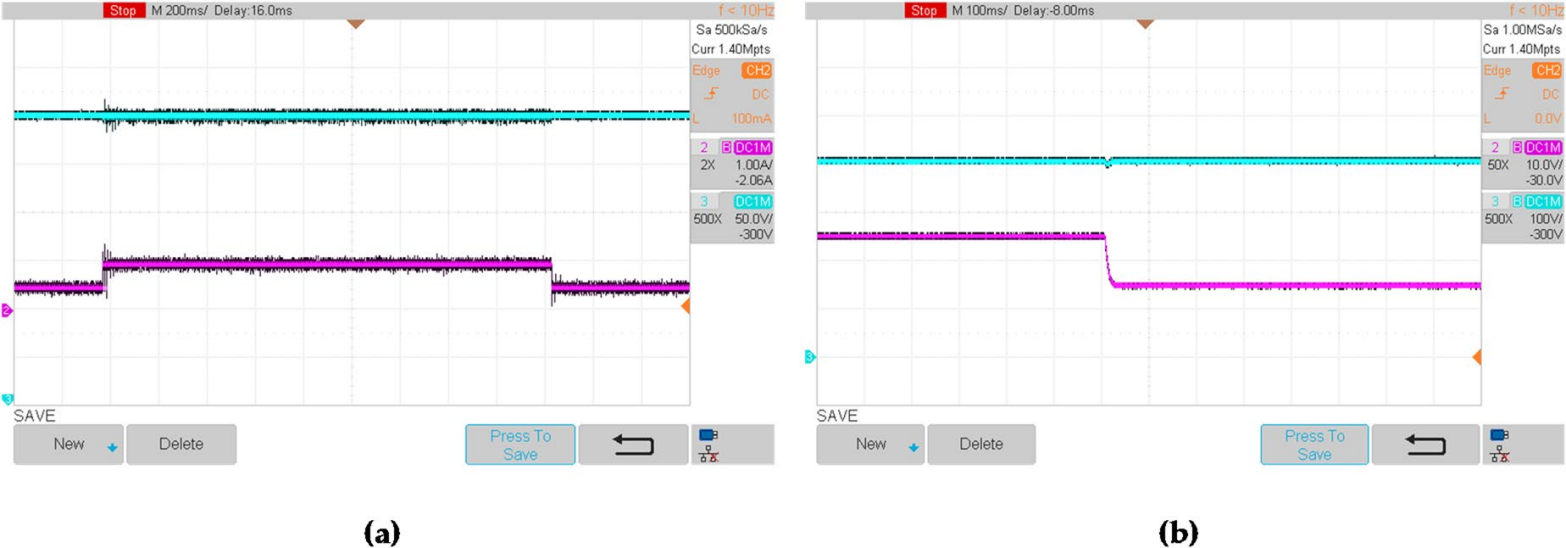
Dynamic response of the proposed converter, (**a**) step change of the load, (**b**) step change of the input voltage.

Closed-loop dynamic tests are presented in Fig. 19a,b, showing the dynamic response under a step load change and a step input-voltage change, respectively. In the first test (Fig. 19a), a step change in load is applied to the converter while the output-voltage regulation loop is enabled. This scenario is particularly important because high-gain converters can be sensitive to sudden load variations due to energy redistribution among inductors and intermediate capacitors. The experimental waveform demonstrates that when the load changes abruptly, the controller effectively compensates by adjusting the duty cycle, so the output voltage remains tightly regulated. In practice, this means the output voltage exhibits only a small deviation (undershoot/overshoot) immediately after the load step, followed by a fast return to the reference value. The transient duration is short, indicating that the closed-loop bandwidth and damping are properly designed, and the system does not suffer from slow recovery or oscillatory behavior. The limited voltage deviation confirms that the controller has adequate disturbance-rejection capability against load disturbances, and the short settling time confirms that the converter’s closed-loop poles are placed appropriately (stable and well damped). Overall, this experiment validates that the proposed control strategy can maintain a nearly constant DC-bus voltage even when the output power demand changes suddenly, which is exactly the operating condition encountered in DC microgrids, motor drives, and pulsed-load applications.

In the second test (Fig. 19b), a step change in the input voltage is applied while the converter remains in closed-loop regulation. This test is highly relevant for renewable energy systems because PV panels and fuel cells naturally experience input-voltage variations caused by irradiation changes, temperature effects, fuel flow dynamics, or upstream converter interactions. The experimental result shows that when the input voltage is disturbed, the controller quickly adjusts the switching command to preserve the regulated output voltage. Similar to the load-step case, the output voltage deviation is small and the response settles rapidly, demonstrating that the controller provides strong line-regulation performance. Importantly, the waveform indicates that the system response is smooth and non-oscillatory, which implies sufficient phase margin and good closed-loop damping. This confirms that the controller is not only stabilizing the converter but also ensuring robust regulation across input-voltage fluctuations an essential requirement for practical renewable-energy interfaces and high-voltage DC bus applications.

From a control-performance perspective, the experimental results confirm three key points. First, the output-voltage controller is effective: it maintains regulation during both load and line disturbances with minimal steady-state error. Second, the transient response is fast: the settling time is short, meaning the converter can recover quickly and keep the DC bus stable. Third, the deviations are small and well-contained: the overshoot/undershoot is limited, indicating that the controller does not overreact and the system is properly damped. These outcomes collectively validate that the selected closed-loop control approach is practical and well tuned for the proposed converter’s dynamics. In other words, the control design is not only theoretically justified by the small-signal modeling and pole-placement framework, but it is also experimentally verified under realistic operating disturbances.

**Fig. 20:**
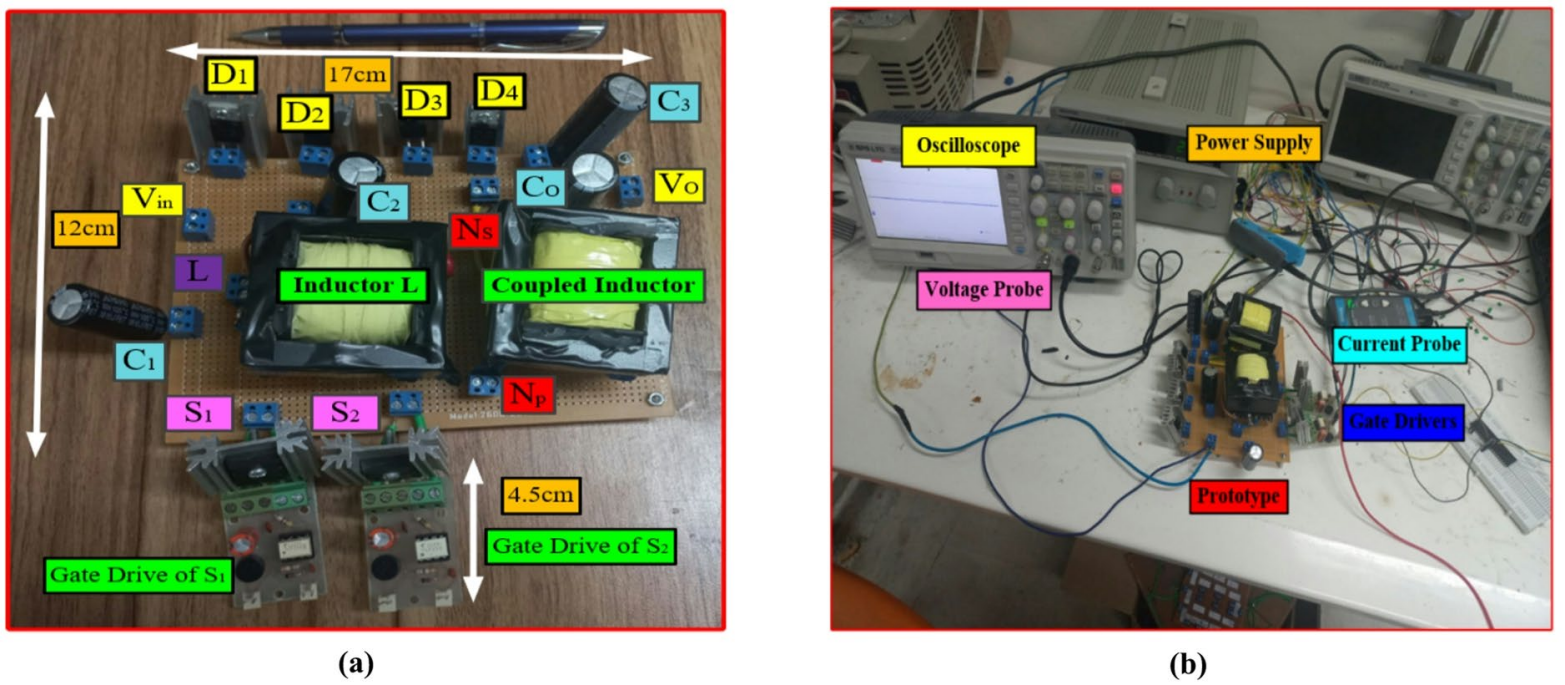
Photographs of (**a**) experimental set-up, (**b**) experimental prototype.

Finally, it is worth emphasizing that these dynamic tests are particularly meaningful for this converter because it includes multiple energy-storage elements (input inductor, magnetizing inductance, and intermediate capacitors) in a quadratic high-gain structure. Maintaining stable regulation in such converters requires a controller that can manage energy redistribution quickly without causing oscillations or excessive voltage stress. The experimental waveforms demonstrate that the proposed controller achieves this goal successfully, supporting the suitability of the converter for DC microgrid DC-bus regulation, renewable-energy front-end interfaces, and industrial DC-link applications. Figure 20a,b show images of the experimental test bench and the constructed hardware prototype of the proposed converter, respectively. These figures illustrate the overall laboratory setup, including the power stage, gate-driving circuitry, and the main measurement instruments used during the experimental validation.

## Conclusions

This work introduces a quadratic boost architecture, tailored to renewable energy system applications, with tightly suppressed input-current ripple. The proposed architecture is benchmarked against 16 recently published topologies (2023–2025). Under a 50% duty cycle with a turns ratio of 2, it delivers a higher voltage gain than all compared designs. Additionally, device stress is kept small: the maximum switch voltage is limited to 25% of the output voltage. The converter offers two independent knobs duty cycle and the coupled-inductor turns ratio for regulating gain, while achieving high output voltage at comparatively low duty cycles. Operating at lower duty reduces switch conduction losses, contributing to higher efficiency. The prototype is rated at 400 W and reaches 95.23% efficiency at full load, outperforming most counterparts in the comparison set. These attributes very high gain, low switch stress, and dual-parameter control make the proposed converter a strong candidate wherever substantial voltage elevation is required in renewable energy system applications.

## Data Availability

All data generated or analyzed during this study are included in this published article.
